# Levels and building blocks—toward a domain granularity framework for the life sciences

**DOI:** 10.1186/s13326-019-0196-2

**Published:** 2019-01-28

**Authors:** Lars Vogt

**Affiliations:** Rheinische Friedrich-Wilhelms-Universität Bonn, Institut für Evolutionsbiologie und Ökologie, An der Immenburg 1, 53121 Bonn, Germany

**Keywords:** Building block, Level, Hierarchy, Domain granularity framework, SEMANTICS, Ontology, Granularity, Knowledge management

## Abstract

**Background:**

With the emergence of high-throughput technologies, Big Data and eScience, the use of online data repositories and the establishment of new data standards that require data to be computer-parsable become increasingly important. As a consequence, there is an increasing need for an integrated system of hierarchies of levels of different types of material entities that helps with organizing, structuring and integrating data from disparate sources to facilitate data exploration, data comparison and analysis. Theories of granularity provide such integrated systems.

**Results:**

On the basis of formal approaches to theories of granularity authored by information scientists and ontology researchers, I discuss the shortcomings of some applications of the concept of levels and argue that the general theory of granularity proposed by Keet circumvents these problems. I introduce the concept of building blocks, which gives rise to a hierarchy of levels that can be formally characterized by Keet’s theory. This hierarchy functions as an organizational backbone for integrating various other hierarchies that I briefly discuss, resulting in a domain granularity framework for the life sciences. I also discuss the consequences of this granularity framework for the structure of the top-level category of *‘material entity’* in Basic Formal Ontology.

**Conclusions:**

The domain granularity framework suggested here is meant to provide the basis on which a more comprehensive information framework for the life sciences can be developed, which would provide the much needed conceptual framework for representing domains that cover multiple granularity levels. This framework can be used for intuitively structuring data in the life sciences, facilitating data exploration, and it can be employed for reasoning over different granularity levels across different hierarchies. It would provide a methodological basis for establishing comparability between data sets and for quantitatively measuring their degree of semantic similarity.

## Background

Arranging a heterogeneous collection of entities into a set of different levels (layers or strata) that are organized in a linear hierarchy from a fundamental level at the bottom to some higher level at the top is a general ordering scheme that dates back at least as far as to ancient times [1]. In biology, attempts to answer the question of how molecules make up cells and cells make up organisms have led to various proposals of compositional hierarchies of different levels of biological organization of living systems and their component parts [2–22].

The underlying levels idea is simple and elegant. It can be flexibly used in many different contexts [23], ranging from descriptions to explanations and the provision of ontological inventories [24]. It is not only frequently used in textbooks [25–27], but also provides an important conceptual framework in various scientific and philosophical debates, including debates on downward causation, mechanistic explanation, complexity, reduction, and emergence [28–32].

Various applications of the levels idea have been proposed in science and philosophy [4, 29, 33–43]. Although distinct from each other, many of them also relate to one another and take subtly different forms when applied in related contexts, which often results in conceptual problems [23]. Oppenheim and Putnam’s [33] theory of reduction, for instance, attempts to explain phenomena of a higher-level science through theories that refer to entities and to theories from the more fundamental science, with the goal of achieving the *unity of science*. As a consequence, however, levels of material entities are associated with levels of broad scientific disciplines (e.g., physical, chemical) and of their corresponding theories and this is a problem, because it leaves the question unspecified, why objects of, for example, physics, which range from sub-atomic particles to entire planets and the universe as a whole, comprise a single level (Bechtel and Hamilton [44]).

Many philosophers have made attempts to establish criteria for the validity or usefulness of the levels idea, sometimes expressed in form of necessary and sufficient formal criteria, but no commonly accepted consensus has been reached for any particular set of criteria [23, 32]. Instead of having to decide and stick with a specific account of levels, Craver ([23], p.2) therefore suggests *descriptive pluralism* about levels, claiming that “the world contains many distinct, legitimate applications of the levels metaphor that are either unrelated or that have only indirect relations with one another.”

Irrespective of the lack of commonly accepted formal criteria, the different accounts of levels suggested so far usually all have in common that each level must represent an increase in organizational complexity, with each entity of a higher level being directly composed of entities belonging to the next lower level [45], resulting in a linear hierarchy of levels from a bottom level to a top level. Moreover, the idea presupposes that entities exist for which it makes sense to understand them as being at the same level.

The idea of levels and of hierarchies based on levels has also been discussed in information science and ontology research. Here, it has become increasingly important due to the continuously growing need of researchers to manage large amounts of data (i.e., *Big Data*) with the help of computers and software applications, resulting in a new driving force for scientific exploration, called *data exploration* or *eScience* [46]. Big Data and eScience bring about the necessity for researchers to communicate biological data via the World Wide Web and to use databases and online repositories to store, document, archive, and disseminate their data. They also require data to be standardized accordingly and to be computer-parsable. All this can be facilitated by the use of ontologies [47–52]. As a consequence, ontology researchers have developed their own approaches to levels, which they call *granularity levels*, and to different types of hierarchies based on levels, which they call *granular perspectives*. Ontology researchers provide explicit criteria for identifying and demarcating different levels and different hierarchies. These criteria specify what is called a *granularity framework*.

In the following, I develop a domain granularity framework for the life sciences that ranges from the atomic level to the level of multi-cellular organisms. The framework attempts to reflect the hierarchical anatomical organization of organisms, marking an important step towards developing a general overarching information framework for the life sciences. Since morphology takes a central role in all attempts of developing a hierarchical system of levels of biological entities, because unambiguously modeling the various granularity relations across morphological entities in a consistent way has been challenging, I focus mainly on morphology. Morphology is also “... one of the covering disciplines that spans [almost] every single entity in any biological organism” ([53], p. 65). It provides diagnostic knowledge and data for many disciplines within the life sciences [54, 55]. And morphological terminology provides the *basic reference system and descriptive framework for the supra-molecular domain in the life sciences*. It is central to all efforts of biological inventorying and to biological knowledge representation in general; and it provides a common backbone for the integration of all kinds of different biological information [47, 48, 56–58].

The paper is divided into two sections. In the first section I briefly discuss a formal approach to levels and hierarchies proposed by ontology researchers, which is based on granular partitions. I compare the notion of a cumulative organization, which most theories of granularity assume for the anatomical organization of biological entities, with the cumulative-constitutive organization and discuss some of the conceptual problems that the latter brings about. I take a brief look at the granularity scheme implicit in the Basic Formal Ontology (BFO), before I introduce the *general theory of granularity* proposed by Keet [59–61] that allows the integration of various different granular perspectives (i.e., hierarchies).

In the other section I discuss BFO’s characterization of bona fide objects based on the identification of different types of causal unity. I suggest adding two more types of causal unity for characterizing functional and historical/evolutionary bona fide entities. I also introduce the concept of *building blocks*, which gives rise to a hierarchy of levels of building blocks that specifies its own granular perspective. This hierarchy is intended to function as an organizational backbone for integrating various additional granular perspectives that are relevant in the life sciences, resulting in a domain granularity framework for the life sciences. I briefly discuss the implicit consequences of this approach for the structure of the top-level category of ‘material entity’ in BFO. I conclude by discussing the suitability of the domain granularity framework here suggested for providing the basis on which an overarching information framework for the life sciences [62] can be developed.

## Methods

### Ontologies and granularity

Information scientists and ontology researchers developed an account of levels that follows a formal approach allowing for computer-parsability and automated reasoning over hierarchies of different levels of granularity, with each hierarchy being understood as a distinct granular perspective. Ontologies play an essential role in this approach. Ontologies, together with other Semantic Web technologies, also play a significant role in reliably communicating and managing data within and between databases and online repositories, providing hierarchies a practical field of application with commercial significance.

An *ontology* consists of a set of terms with commonly accepted definitions that are formulated in a highly formalized canonical syntax and standardized format, with the goal to yield a lexical or taxonomical framework for knowledge representation [63]. The terms are organized into a nested hierarchy of classes and subclasses, forming a tree of increasingly specialized terms that is called a *taxonomy* [64]. However, when ontology researchers need to refer to hierarchies other than taxonomies, for example, a *partonomy* (i.e., a hierarchy based on part-whole relations), they usually do that in reference to some (external) granularity framework. Such partonomies, however, are usually only expressed indirectly through formalized descriptions specifying particular parthood relations between resources within the taxonomy of an ontology. This often results in the respective ontology containing several disconnected partonomies that provide only locally applicable parthood-based granularity schemes, as opposed to a single globally and universally applicable scheme.

Whereas the number of biomedical ontologies is continuously increasing [65], they often differ considerably, and their taxonomies as well as their implicit partonomies and even some of their term definitions are often inconsistent across each other [66–68]. As a consequence, if databases and online repositories differ with respect to the ontologies they use, their contents are likely to be incomparable, which significantly hampers data exploration and integration. A solution to this problem involves two distinct approaches: using *formal top-level ontologies* [66, 69] such as BFO [70, 71] and applying a *general formal theory of granularity* for developing a domain granularity framework that can be applied as a meta-layer across various ontologies.

### Partial order, granular partition, and granularity tree

Key to the development of any formal theory of granularity is the formal characterization of the relation that holds between entities belonging to different levels of granularity. A first step is to identify partial order relations. In mathematics and logics, a *partial order* is a binary relation ‘R’ that is *transitive* (if *b* has relation R to *c* and *c* has relation R to *d*, than *b* has relation R to *d*: (R*bc*) (R*cd*) → R*bd*), *reflexive* (*b* has relation R to itself: R*bb*), and *antisymmetric* (if *b* has relation R to *c* and *c* has relation R to *b*, than *b* and *c* are identical: (R*bc*) (R*cb*) → *b* = *c*) [72]. An example of a partial order relation is the parthood relation.

*Granular partitions* are based on partial order relations [73–76]. Granular partitions are involved in all kinds of listing, sorting, cataloging and mapping activities. A granular partition is a hierarchical partition that consists of cells (here used in the general non-biological meaning of cell) that contain subcells. It requires a specific theory of the relation between its cells and subcells: (i) the subcell relation is a partial ordering relation; (ii) a unique maximal cell exists that can be called the root cell; (iii) chains of nested cells have a finite length; and (iv) if two cells overlap, then one is a subcell of the other, therewith excluding partial overlap [73–76]. An empirically meaningful theory of granular partition also requires a theory of the relations between cells of the partition and entities in reality (i.e., projective relation to reality [73–75]).

Depending on what is partitioned and the ontological nature of the parts, one can distinguish a bona fide granular partition from a fiat granular partition. A bona fide *granular partition* partitions a bona fide object (i.e., an entity that is demarcated by a bona fide boundary and thus exists independent of any human partitioning activities) into its bona fide object parts. A *fiat granular partition* partitions any material entity into its fiat entity parts (i.e., entities that are demarcated by a *fiat* boundary and thus exist as a consequence of human partitioning activities) (for a distinction of bona fide and fiat entities see discussion below and [70, 71, 77]).

A granular partition can be represented as a tree, with the nodes and leaves of the tree being the granular parts. This tree is called a *granularity tree* [69, 76, 78]. Every finite granular partition can be represented as a rooted tree of finite length [74, 75, 79–81]. In a granularity tree, a granularity level is a *cut* (sensu [82]; see Fig. 2b) in the tree structure. Within a granularity tree, different levels of granularity can be distinguished, with the root being a level itself, and all immediate children of the root another level, etc. The elements forming a granularity level are *pairwise disjoint*, and each level is *exhaustive*, because for every entity *b* of the partition exists some other entity *c* of the same partition, which belongs to another level of granularity, and *b* stands in a partial ordering relation to *c*, or vice versa [76]. If the partitioning relation is a mereological relation such as the part-whole relation, all entities belonging to one granularity level in a granularity tree exhaustively sum to the whole (i.e., the root cell) that is partitioned [76].

Partitioning relations possess constrains regarding the type of entities that they partition. The primitive part-whole relation, for instance, exists only between instances (particulars/individuals) [23, 83–85] (for a translation to a class expression of parthood see [83, 86]). As a consequence, parthood-based granular partitions can be represented as *instance granularity trees*. The class-subclass relation is also a partial ordering relation. However, it exists only between types (classes, universals). Granular partitions based on a class-subclass relation therefore can be represented as *type granularity trees*. The taxonomy of terms of an ontology represents such a type granularity tree. (see also instance and type granularity tree in [58, 87]).

Hierarchies are based on *strict partial ordering* relations, which represent *irreflexive* (*b* cannot stand in relation R to itself: ¬R*bb*) partial ordering relations. As a consequence, hierarchies represent a specific case of granular partitions and granularity trees. The direct proper parthood relation is a strict partial ordering relation. This complies with any formal system of minimal mereology, including pure spatiotemporal parthood.

### Biological reality: the problem with the cumulative constitutive hierarchy

**Fig. 1:**
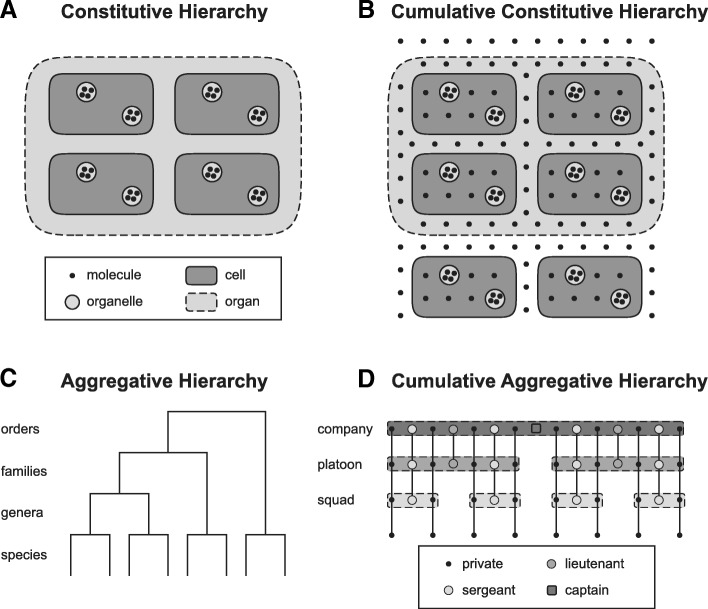
Four different Types of Hierarchies. **a** A constitutive hierarchy of molecules, organelles, cells, and organs of a multi-cellular organism. It can be represented as an encaptic hierarchy of types, with every molecule being part of some organelle, every organelle part of some cell and every cell part of some organ. **b** The same set of entities as in A), organized in a cumulative constitutive hierarchy, which models the organization of biological material entities more accurately. Here, not every molecule that is part of an organism is also necessarily part of some organelle and not every cell necessarily part of some organ. **c** An aggregative hierarchy is based on mereological/meronymic inclusion that results from a part-whole relation (e.g., ecological hierarchies [15, 17]) or it is based on taxonomic inclusion [138] that results from a subsumption relation (e.g., Linnean taxonomy). In case of mereological inclusion, this hierarchy represents a mereological granularity tree and higher level entities consist of parts that are not physically connected, but only associated with each other. **d** In a cumulative aggregative hierarchy, as it is used in the hierarchical organization of military stuff, individuals with higher ranks, such as sergeants, lieutenants, and captains, appear in aggregates of higher order, so that squads consist of privates and sergeants, in the next level platoons of privates, sergeants, and lieutenants, and companies of privates, sergeants, lieutenants, and captains. (*Figure modified from* [58])

On the basis of the characterization of hierarchies mentioned above one can distinguish four basic types of hierarchical systems [17, 21, 88]: (i) constitutive hierarchies, (ii) cumulative constitutive hierarchies, (iii) aggregative hierarchies, and (iv) cumulative aggregative hierarchies (Fig. 1), of which only the former two hierarchies are of interest in the here discussed context. Interestingly, constitutive hierarchies are commonly used by philosophers and ontology researchers to model granularity, whereas biologists use cumulative constitutive hierarchies.

In a *constitutive hierarchy* [38], all material entities of a given level of granularity constitute the entities of the next coarser level. For instance, aggregates of all atoms that exist constitute all molecules that exist and aggregates of all molecules constitute all cells [17]. In other words, coarser level entities consist of physically joined entities of the next finer level of granularity [88]. A constitutive hierarchy is thus based on partonomic inclusion resulting from an *irreflexive* proper part-whole relation, with bona fide entities of different levels of granularity being mereologically nested within one another, thus representing a *mereological granularity tree* [76].

Most granularity schemes suggested in the ontology literature so far presuppose a constitutive organization of material entities [78, 89] (for an exception see [58]), and many bio-ontologies, although often not accompanied by an explicit representation of formally defined levels of granularity, also follow this scheme. This is problematic given that constitutive hierarchies not only assume that coarser level entities always exclusively consist of aggregates of entities of the next finer level, but also that every entity belonging to one level of granularity is part of some entity of the next coarser level of granularity (Fig. 1a). Unfortunately, this is not the case for many material entities: ions or chlorine radicals demonstrate that not every atom necessarily is part of a molecule; in humans, extracellular matrix (ECM; a macromolecular formation that is *not* a component of cells, but a component of tissues and therefore also organs and multi-cellular organisms) and blood plasma demonstrate that not every molecule is part of a cell; protozoa, protophyta, erythrocytes, coelomocytes, or leukocytes demonstrate that not every cell necessarily is part of an organ [87]. Obviously, *not all* the entities belonging to one level of granularity necessarily form parts of entities of the next coarser level.

Moreover, constitutive hierarchies also assume that all parts of any given level of granularity exhaustively sum to their complex whole (Fig. 1a). Regarding biological material entities this implies that the sum of all cells of a human individual would have to yield the human individual as a whole. The totality of cells of any given human being, however, does not sum to the body as a whole, since this mereological sum would not include the ECM in which the cells are embedded and which provides the topological grid that determines the relative position of the cells to one another. The aggregation of cells would disintegrate without the ECM and could not constitute the body as a bona fide whole. Moreover, since not all atoms are part of a molecule and not all subatomic particles are part of an atom, neither the sum of all molecules, nor the sum of all atoms that exist in the universe at a given point in time exhaustively sum to the universe as a whole [87]. As a consequence, not all parts that share the same granularity level necessarily exhaustively sum to the maximal whole (contradicting [76, 78]).

**Fig. 2:**
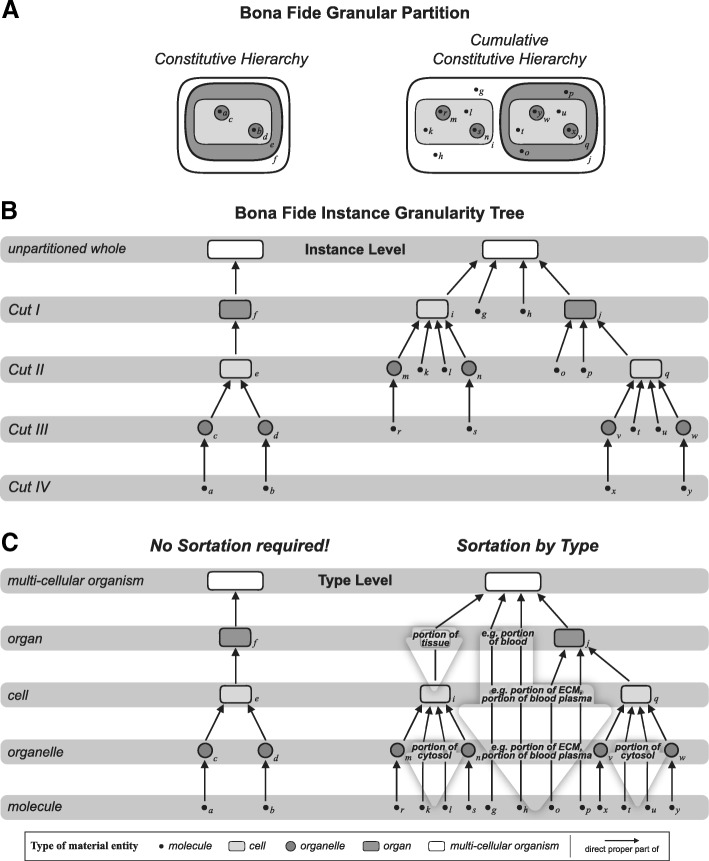
Instance Granularity Tree and Type Granularity Tree based on bona fide Granular Partition for Constitutive and Cumulative Constitutive Hierarchies. **a** Compositional partitions of a constitutively and a cumulative-constitutively organized idealized multi-cellular organism into their constitutive bona fide object parts. Four corresponding partitions are shown. Left: into organs (f); cells (e); organelles (c, d); and molecules (a, b). Right: into organs with cells and extracellular molecules (i, j, g, h); cells with organelles and extracellular and cellular molecules (q, m, n, o, p, k, l); organelles and molecules (v, w, t, u, r, s); and molecules (x, y). **b** The four compositional partitions from A) represented as a bona fide instance granularity tree. Each partition constitutes a cut in the instance granularity tree (Cut I–IV) and thus an instance granularity level. **Left**: Instances of the same type of material entity do not belong to different cuts and thus are restricted to the same level of instance granularity. **Right**: Instances of the same type of material entity, for instance ‘molecule’, belong to different cuts and therefore to different levels of the respective instance granularity tree. The extension of the class ‘molecule’ thus transcends the boundaries between instance granularity levels. **c Left**: The bona fide instance granularity tree can be directly transformed into the corresponding type granularity tree—no sortation of any parts across the boundaries of granularity levels required, because the topology of the bona fide instance granularity tree is identical with the bona fide type granularity tree. **Right**: The bona fide instance granularity tree cannot be directly transformed into or mapped upon the corresponding type granularity tree. However, by following the simple and intuitive rule that a type must occupy the same granularity level as its finest grained instance (i.e., *sortation-by-type* [58]) and by applying the concept of *granular representation* (see further below), one can transfer the instance granularity tree into a corresponding type granularity tree. (*Figure modified from* [87])

Instead of employing a constitutive hierarchy, biologists have argued that typical biological material entities such as multi-cellular organisms are organized according to a *cumulative constitutive hierarchy* [17, 21, 88] (Fig. 1b). When comparing the characteristics of constitutive hierarchies with those of cumulative constitutive hierarchies one can easily see why most approaches to granularity that are frequently used in ontologies, but also the formal theory of granularity of Kumar et al. [78], model the bio-medical domain on the basis of a constitutive hierarchy. When partitioning a particular multi-cellular organism (i.e., unpartitioned whole, Fig. 2b) into its direct proper bona fide parts according to a constitutive hierarchy, *all* the parts belonging to a cut, and thus to an instance level, instantiate the same basic type of anatomical entity (Fig. 2b, left). Therefore, each cut in the instance granularity tree can be associated with a specific basic type of anatomical entity. As a consequence, instead of talking about ‘Cut I’, one could just as well talk about the ‘organ’ granularity level. Translating or mapping the topology of an instance granularity tree to its corresponding type granularity tree is thus straight forward and poses no conceptual problems—*if* one applies a constitutive hierarchy for partitioning the multi-cellular organism that is (Fig. 2c, left). One could even derive a globally applicable, linear compositional levels hierarchy for the life sciences. One would only have to apply the constitutive hierarchy model and compare the type granularity trees of several multi-cellular organisms across various taxa.

However, when applying the cumulative constitutive hierarchy model, the entire process becomes more complex and conceptually more challenging [58, 87]. According to the cumulative constitutive hierarchy, the parts of a multi-cellular organism that belong to a cut of an instance granularity tree do *not all* instantiate the same basic type of anatomical entity (Fig. 2b, right). For instance, the parts that belong to the first cut in the example shown in Fig. 2b instantiate organs, cells, and molecules. As a consequence, the mereological sum of all entities belonging to one instance granularity level does *not necessarily* sum to the unpartitioned whole (see, e.g., ‘Cut III’ in Fig. 2b, right). Thus, one must conclude that Kumar et al.’s [78] theory of granularity and one of Reitsma and Bittner’s [76] criteria for mereological granularity trees are not conformant with anatomical reality [58].

Moreover, the topology of the resulting instance granularity tree cannot be easily translated into its corresponding type granularity tree, because each instance level comprises different types of entities (except for the root and the finest level). A consequence of cumulative constitutive hierarchies is that, when partitioning a multi-cellular organism, different instances of the same basic type of anatomical entity can belong to different instance granularity levels. In other words, when conceiving types of anatomical entities as classes, the extension of a class such as ‘bio-molecule’ crosses the boundaries of different levels of instance granularity when applying the cumulative constitutive hierarchy. Therefore, mapping types directly to instance levels would result in some types being associated with more than one level.

This poses a fundamental problem, because ontologies are dealing with types (i.e., classes) and not with individuals (i.e., instances), and thus require a type-based granularity framework. I have proposed an intuitive solution, i.e., *sortation-by-type*, in which a type granularity tree is derived from an instance granularity tree by ranking types according to the lowest level of granularity of their corresponding instances [58]. Sortation-by-type can be seen as a sort of *granular sedimentation* of all instances of one type to the lowest level they occupy (see large transparent arrows in Fig. 2c, right). Whereas this approach seems to be intuitive, the downside is that in the type granularity tree, the entities belonging to a granularity level neither exhaustively sum to their respective whole (except for the lowest level), nor do all of them form parts of the entities belonging to the next higher granularity level [58].

### The granularity scheme implicit in the basic formal ontology

Formal top-level ontologies such as BFO [70, 71] play a key role in establishing standards across different ontologies. BFO provides a genuine upper ontology upon which all ontologies of the Open Biomedical Ontologies Foundry (OBO Foundry [57, 90]) are built. Together with the OBO Relations Ontology it is one of the guarantors for the interoperability of the ontologies within OBO Foundry.

**Fig. 3:**
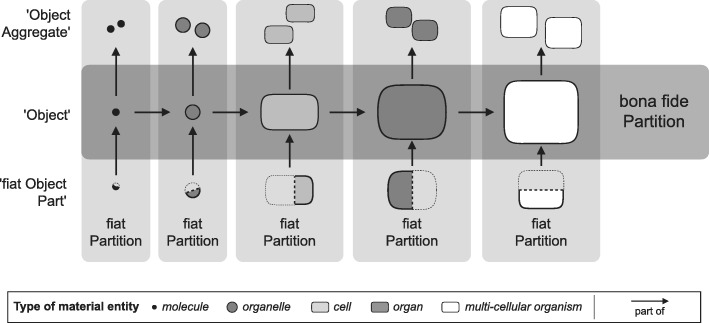
BFO’s Basic Granularity Framework. A bona fide partition from a multi-cellular organism to a molecule represents the center of BFO’s granularity framework and reflects direct subclasses of BFO’s ‘object’ for the biological domain. According to BFO, each level of the corresponding bona fide granularity tree must be modeled by its own domain reference ontology (i.e., a molecule ontology, a cell ontology, etc.). Within each such level-specific ontology, BFO’s top-level distinction of ‘object’, ‘fiat object part’, and ‘object aggregate’ indicates a basic fiat partition that orthogonally crosses the bona fide partition. The bona fide partition can therefore be understood as an integrating cross-granular backbone for the different ontologies of a given domain together with their implicit fiat partitions

Because BFO is an upper ontology, its taxonomy is comparably flat and does not include any distinction of different granularity levels of material entities. However, BFO’s distinction of ‘object’, ‘object aggregate’, and ‘fiat object part’ as top-level categories of ‘material entity’ [70, 71] can be interpreted as a basic granularity scheme applied for modeling the granularity within a given level of object granularity. The underlying basic idea is that a certain domain first must be partitioned into its top-level object categories, resulting in a domain-specific bona fide granularity tree (i.e., a granularity tree that is based on bona fide granular partitions; see [76]), e.g., ‘bio-macromolecule’ < ‘organelle’ < ‘cell’ < ‘organ’ < ‘organism’. According to BFO, in order to comprehensively cover the domain, each level of this bona fide granularity tree must be modeled by its own level-specific domain reference ontology, with cross-ontology relations managing the relationships between entities of different levels. Therefore, in a next step, the distinction of ‘object’, ‘fiat object part’, and ‘object aggregate’ indicates within each such ontology a simplified model for fiat partitions and fiat granularity trees (see Fig. 3). Of course, object aggregates can be parts of larger object aggregates and fiat object parts can be further partitioned to smaller fiat object parts, thereby extending the basic scheme shown in Fig. 3 with additional levels.

This approach to modularizing granularity, however, does not seem to be very practicable, because it implies that instead of developing a single anatomy ontology of a specific taxon of multi-cellular organisms, one would have to (i) develop several granularity-specific ontologies, ranging from an ontology for molecules, to an ontology for organelles, for cells, for tissues, for organs and for body parts for this specific taxon, and (ii) one would have to develop an additional layer of axioms and relationships to define the granularity relations between entities across these different ontologies.

Because BFO does not provide a formal granularity framework, applying the sub-categories of ‘material entity’ (i.e., ‘object’, ‘fiat object part’, and ‘object aggregate’) can be ambiguous. As a consequence, many of the currently available biomedical ontologies within OBO Foundry significantly vary regarding their underlying granularity assumptions and their top-level class-structure for ‘material entity’ (see subclasses of, e.g., ‘material anatomical entity’ of CARO, ‘anatomical structure’ of HAO, ‘material entity’ of OBI, ‘plant structure’ of PO, ‘anatomical structure’ of ZFA, ‘independent continuant’ of CL, ‘cellular component’ of GO). One could argue that BFO fails to provide a top-level structure for ‘material entity’ that can be applied for modeling the various domains covered by OBO Foundry ontologies. This causes problems with the comparability of biomedical ontologies and substantially limits the comparability of data across databases and online repositories that reference these ontologies. The life sciences in general and comparative morphology in particular, but also the *compositional biology* style of biological theorizing [91], would benefit from a consistent granularity framework that is grounded in reality and that accounts for the organizational complexity of anatomy. In order to allow algorithm-based reasoning and inferencing, such a framework requires an underlying formal theory of granularity that explicitly states formal granularity relations and explicitly ranks levels of granularity. Unfortunately, most anatomy ontologies are only based on implicit assumptions regarding granularity.

### Keet’s formal theory of granularity

Keet [59–61] has developed a formal theory of granularity that is agnostic regarding cumulative or cumulative constitutive hierarchies and thus circumvents some of the problems of theories of granularity that have been proposed by others (e.g., [78]; problems discussed in [58]). Keet [61] argues that granularity always involves modeling something according to certain criteria, with each model together with its criteria defining a *granular perspective*. Finer levels within a perspective contain knowledge or data that are more detailed than the next coarser level, and coarser levels of granularity simplify or make indistinguishable finer-grained details. A particular granularity level, however, must be contained in one and only one granular perspective, whereas a particular entity (individual or type) may reside in more than one level of granularity, but all levels in which it is contained must belong to distinct granular perspectives [92]. Moreover, a granular perspective has at least two levels of granularity and there has to be a strict total order between the entities of different levels of a given perspective. And if there is more than one granular perspective for a subject domain, then these perspectives must have some relation between each other. This way, several different perspectives of granularity, each with its granularity tree and its corresponding set of granularity levels, can coexist within the same granularity framework. For instance, a granular perspective of relative location that is based on fiat granular partitions, alongside with a granular perspective of structural composition that is based on bona fide partitions, a perspective of biological processes that is based on temporal parthood relations (i.e., processes partitioned into their sub-processes), a perspective of functional units that is based on functional parthood relations (i.e., functional units partitioned into their functional sub-units), and a granular perspective based on developmental relations [58].

The idea that a domain can be modeled by different granular perspectives is not new [69, 88, 91, 93, 94], but Keet [61] provides the first formal theory of granularity that incorporates different granular perspectives within a single *domain granularity framework*. Therefore, Keet’s theory can be understood as an attempt to accept descriptive pluralism about the idea of levels [23]. However, it also represents an attempt to integrate the resulting set of diverse hierarchies within an integrated and strictly formalized framework, her *general formal theory of granularity*.

A granular perspective can be specified by the combination of a *granulation criterion* (*what to granulate*) and a specific *type of granularity* (*how to granulate*) (for a detailed discussion see [61]). When applied to a corresponding object, a granular perspective partitions the object resulting in a specific type of granularity tree. Each perspective has exactly one granulation criterion and exactly one type of granulation. This combination determines the uniqueness of each granular perspective. All granular perspectives contained in a domain are thus disjoint. Keet [61] presumes that a domain of reality can be granulated according to different types of granularity (mechanisms of granulation), requiring the existence of a certain type of *granulation relation* that must be specific to each particular granular perspective. The entities (individuals or types) granulated by a type of granularity are disjoint.

Various different types of granulation relations can be applied, which can be classified into (i) scale-dependent (e.g., resolution, size) and (ii) non-scale-dependent types of granularity (e.g., *mereological parthood*: structural parthood, functional parthood, spatial parthood, involvement; *meronymic parthood*: membership, constitution, sub-quality relations, participation) [61, 95]. Within a given perspective, the granulation relation relates entities of adjacent granularity levels with one another. If a granular perspective has more than two levels of granularity, the granulation relation must be *transitive*. If a granulation relation is intransitive, then the respective perspective has only 2 levels.

The granulation criterion delimits the kind or category of properties according to which the domain is partitioned, the levels identified, and the subject domain granulated (i.e., data, information, or knowledge). It specifies an aspect that all entities in a granular level must have in common, whereas the contents of a level can be either entity individuals (i.e., instances) or types (i.e., universals, classes), but not both. It comprises either (i) at least two properties, none of which is a quality property (for non-scale-dependent types of granularity) or (ii) at least one property that is not a quality property together with exactly one quality property that has a measurable region (for scale-dependent types of granularity) [61].

Keet’s [61] formal theory of granularity thus provides the respective formal definitions, axioms, and theorems that allow the formal representation of granular partitions based on parthood relations (i.e., mereology) as well as on taxonomic inclusion (i.e., class-subsumption hierarchies based on set theory) and other types of granulation relations [60]. It even accommodates both quantitative (i.e., arbitrary scale) and qualitative (i.e., non-scale-dependent) aspects of granularity.

Keet’s theory of granularity also provides a well suited framework for analyzing and identifying some of the problems of some of the granularity schemes that have been proposed earlier, taking Eldredge’s somatic hierarchy [9] as an example—this criticism applies to many of the published levels schemes, even including Kumar et al.’s [78] scheme: The somatic hierarchy comprises an ‘atom’, ‘molecule’, and ‘cell’ level together with an ‘organelle’, ‘organ’, and ‘individual organism’ level of granularity. An obvious problem of this hierarchy is that its underlying granulation criterion has been conflated between levels, because spatio-structural entities have been mixed with functional entities. As a consequence, the underlying granulation relation varies depending on the level between spatio-structural parthood and functional parthood. Moreover, the ‘tissue’ level seems to involve a scale-dependent granularity type, because it concerns resolution—a tissue is the representation of a cell aggregate at a coarser level of resolution, in which the finer-grained details of the cell aggregate that enable the individuation of individual cells are simplified or made indistinguishable. This mixing of criteria and types of granularity results in inconsistent granulation: a mono-cellular organism is an entity that belongs to both the ‘cell’ and the ‘individual organism’ level of the same perspective, but according to Keet [61] an entity can only reside in more than one level if these levels belongs to different granularity perspectives.

## Results

### Developing a domain granularity framework for the life sciences

The increase in formalism coupled with the increase in generality compared to other theories of granularity results in more flexibility and therefore a broader applicability of Keet’s theory. Her theory allows for detailed and sophisticated modeling of a domain by assigning specific types or individuals of entities to specific types of granular perspectives (i.e., hierarchies) that are interconnected and integrated in a common domain granularity framework. This framework can be used (i) as template for the organization of top-level categories of different domain ontologies and (ii) to provide an independent overarching information framework that functions like an additional organizational layer, i.e., a meta-layer, to which terms/resources of different ontologies can be mapped. This meta-layer would provide a consistent and integrated system of well integrated granular perspectives that allows for modeling not only parthood-based hierarchies, but all kinds of other relevant hierarchies, for instance, hierarchies based on developmental or evolutionary relations. It can be formally added onto an existent knowledge base to facilitate the construction of a realism-based and more detailed model of the biological domain (see also [58]).

In order to be broadly applicable throughout many existing bio-medical ontologies, such a domain granularity framework for the life sciences would have to be developed in reference to BFO and its implicit granularity scheme using a *compositional* bona fide *‘object’ granular perspective* that granulates bona fide ‘object’ entities according to a *direct proper parthood* granulation relation (see Fig. 3). All additional granular perspectives can be directly or indirectly related to this compositional perspective, which functions as an organizational backbone for the entire framework, because each additional perspective possesses some level that shares entities with some level of this compositional perspective. The development of such a domain granularity framework, however, may result in new demands that BFO (or some intermediate domain reference ontology) must meet, which could result in the necessity to adapt or extend BFO accordingly.

### Integrating BFO and frames of reference in a domain granularity framework

#### Frames of reference and BFO’s ‘object’ category of ‘material entity’

Smith et al. [71] (see also [70]) characterize BFO’s bona fide *‘object’* category and thus natural units that exist independent of human partitioning activities as causally relatively isolated [96, 97] entities that are both structured through and maximal relative to a certain *type of causal unity*. They distinguish three types of causal unity:

1) *Causal unity* via *internal physical forces*, which unifies an entity through physical forces (e.g., fundamental forces of strong and weak interaction, covalent bonds, ionic bonds, metallic bonding) that are strong enough as to maintain the structural integrity of the entity against the strength of attractive or destructive forces from its ordinary neighborhood. Whereas Smith et al. [71] mention only examples of physical forces that apply to the atomic and molecular scale (atoms, molecules, portions of solid matter such as grains of sand and lumps of iron), I would explicitly include all kinds of physical connections between material component parts, independent of their scale, including cell-cell connections, but also screws, glues, and bolts. Ultimately, they all go back to the physical forces discussed in Smith et al. [71].

2) *Causal unity* via *physical covering* unifies an entity through a common physical covering, for instance, a membrane. This covering may have holes, but must be completely connected (in the sense that a continuous path can be traced between any two points on the surface and that path has no gaps and does not leave the surface) and must still serve as a barrier for entities from inside and entities from outside that are above a certain size threshold. Examples: organelles, cells, tissues, organs.

3) *Causal unity* via *engineered assembly of components* unifies an entity through screws, glues and other fasteners. Often, the parts are reciprocally engineered to fit together (e.g., dovetail joints, nuts and bolts). Examples: cars, ballpoint pens, houses, shoes, power grids.

These three types of causal unity are ontologically not independent from one another, because the latter two *existentially depend* and thus supervene on causal unity via internal physical forces [98]. Moreover, they do not cover all cases of causal unity relevant in the life sciences (BFO does not claim completeness regarding the list of cases of causal unity; see [70, 71]), but are confined to a synchronic approach to causal unity that is associated with a spatio-structural frame of reference (see below). Functional units and historical/evolutionary units are not covered, although they are bona fide entities in their own right that exist independent of any human partitioning activities [77]. In this context it is important to note that functional and historical/evolutionary units are not associated with a spatio-structural frame of reference and are thus not necessarily also spatio-structural units. Moreover historical/evolutionary units are not confined to a diachronic instead of a synchronic causal unity. Diachronic causal unity identifies natural units based on shared historical/evolutionary origin (for a detailed discussion see [77]). Therefore, I suggest two additional types of causal unity that are suited to cover the missing cases:

*Causal unity* via *bearing a specific function* unifies an entity through the function that the entity bears, with its functional component parts bearing sub-functions [98]. This type of causal unity is more general than causal unity via engineered assembly of components and thus includes it.

*Causal unity* via *common historical/evolutionary origin* unifies an entity through the common historical/evolutionary origin of the entity’s component parts. A historical/evolutionary unit is demarcated so that all of its component parts share the same historical/evolutionary origin, with no material entity not belonging to it sharing the same origin [98]. As a consequence, historical/evolutionary units can be spatio-structurally scattered entities such as twins living in different cities or apples from the same tree sold in different supermarkets.

Moreover, because a given material entity can depend on several different types of causal unity at the same time, of which not all are relevant in every context, each type of causal unity is connected to a specific basic frame of reference [98]. Both causal unity via internal physical forces and causal unity via physical covering, at least as conceived by Smith et al. [71] (see also [70]), are associated with a *spatio-structural frame of reference*. A motivation for applying a spatio-structural frame of reference lies in inventorying *what is given* in a particular point in time by focusing on the spatio-structural properties of a given entity (spatio-structural perspective [77]). Causal unity via bearing a specific function, on the other hand, is associated with a *functional frame of reference*, which may be applied for making reliable predictions of *what can happen* in the future by focusing on dispositional/functional aspects of reality (predictive perspective [77]). And causal unity via common historical/evolutionary origin is associated with a *historical/evolutionary frame of reference*, which may be applied for making reliable retrodictions of *what has happened* in the past by focusing on using a set of known types of repeatable processes to reconstruct the sequence of events that may have lead to the currently observable situation (retrodictive (diachronic) perspective [77]).

Because BFO’s general granularity scheme associates to each top-level category of ‘object’ a corresponding ‘fiat object part’ and ‘object aggregate’ category (e.g., ‘molecule’ with ‘fiat molecule part’ and ‘molecule aggregate’) and because we can distinguish different spatio-structural categories of ‘object’ (e.g., ‘atom’, ‘molecule’, ‘organelle’), we can differentiate additional spatio-structural sub-frames of reference, one for each spatio-structural top-level category of ‘object’ that we can distinguish (e.g., ‘atomic frame’, ‘molecular frame’, ‘organelle frame’). Each such frame of reference includes not only the entities of the respective ‘object’ category, but all entities of corresponding ‘fiat object part’ and ‘object aggregate’ categories. One of the reasons for distinguishing different spatio-structural frames of reference lies in enabling the identification of what is comparable in a particular point in time by focusing on entities belonging to a particular top-level ‘object’ category and its corresponding fiat object part and object aggregates entities. As a consequence, the number of spatio-structural frames of reference directly depends on the number of top-level spatio-structural ‘object’ categories we can distinguish.

#### The basic Organization of a Domain Granularity Framework for the life sciences

As a consequence of the relevance of the different cases of causal unity for the life sciences, a domain granularity framework for the life sciences would have to cover three basic categories of granular perspectives: granular perspectives relating to (i) spatio-structural, (ii) to functional, and (iii) to historical/evolutionary material entities. In analogy to BFO’s general granularity scheme discussed above, each such basic category will include one or more corresponding bona fide granular perspectives, with each granularity level of a bona fide perspective having associated ‘fiat object part’ and ‘object aggregate’ fiat perspectives. As a consequence, the number of granular perspectives for each such category depends on the number of granularity levels of its corresponding bona fide perspectives, with each bona fide level requiring some additional associated fiat perspectives.

However, since each of the three basic categories of perspectives corresponds with one of the three basic frames of reference relevant to the life sciences, any given material entity always belongs to *at least* three different granular perspectives—one for each basic frame of reference (i.e., spatio-structural, functional, historical/evolutionary). Moreover, when considering that at least the basic spatio-structural frame of reference actually consists of a set of several distinct spatio-structural frames of reference, one for each identified spatio-structural top-level ‘object’ category, any given material entity actually belongs to *more than three* granular perspectives. In other words, an entity belonging to some level of functional granular perspective will always also belong to some level of historical/evolutionary granular perspective and some level of each of the different spatio-structural granular perspectives, and vice versa. And because all the different granular perspectives of one category overlap in the sense that no granular perspective exists that does not overlap directly or indirectly with the bona fide perspective of this category, the perspectives of the three categories overlap each other as well, thus integrating all the different perspectives of the domain granularity framework. As a consequence, assuming that only one bona fide perspective exists for each basic frame of reference, the bona fide perspectives function as the organizational backbone of the entire framework. Ideally, these bona fide perspectives would directly overlap with each other, which would substantially increase the overall integration of the framework.

### 1st step: Identifying the organizational backbone granular perspective for the life sciences based on building blocks

#### Building block systems: An evolutionary systems-theoretical perspective

Are hierarchies artifactual and thus mind-dependent constructs? If we use the levels idea merely because it takes a central role in our representations of reality, why should we bother to ask nature which hierarchy is most realistic? Whereas these questions are legitimate, evidence exists that suggests that evolution (including cosmic evolution [99]) leads to modularization. If evolution has the tendency to aggregate material entities to larger compositions with a significant increase in complexity, robustness, and stability, resulting in a modularization of matter, then hierarchy is a necessary consequence of evolution. If *building block systems* evolve, which become parts of larger building block systems, then a hierarchical composition of building block systems must result that has lower-level building block systems as its parts. The resulting compositional hierarchy of building block systems is the product of natural processes and thus exists independent of any human partitioning activities.

The idea that evolution has the tendency to evolve such building block systems is not new. Simon [29] argued for the evolution of complex forms from simple ones through purely random processes, with the direction towards complex forms being provided by their stability (“survival of the fittest—i.e., of the stable”, [29], p. 471). Simon argued that “[t]he time required for the evolution of a complex form from simple elements depends critically on the numbers and distribution of potential intermediate stable forms” ([29], p. 471). Hierarchy would thus emerge almost inevitably through evolutionary processes for the simple reason that hierarchical structures are stable [29].

Our understanding of how morphological structures evolve and how they develop during morphogenesis has substantially improved since Simon proposed the idea of building block systems and it seems to support his idea. Especially with the newly emerged field of evo-devo and the discovery of hox genes, we start to understand how regulatory gene networks function like modular structures [100–102] that can recombine with other modules in the course of evolution to form new networks [103], and how they strongly affect development of morphological structures, their evolutionary stability, and their evolvability [104–107]. Some gene regulatory networks have been identified that have the role of individualizing parts of the body during development, and it seems to be the case that these *Character Identity Networks* (ChINs, [105]) are more conserved than are other aspects of character development and thus represent prime candidates for building block systems.

#### Building blocks as Spatio-structural bona fide objects

Taking the idea of building block systems as a starting point, I provide a specific characterization of *building block* as a Lego-brick-like entity that evolves, diversifies, and provides reality’s inventory of basic categories of material entities. The concept of building blocks then provides the basis for a specific account of levels. According to this account, various types of building blocks emerged during evolution, starting when there were only elementary particles present, to a universe that has gradually evolved with the emergence of more and more new types of building blocks [18, 29, 108–112]. This evolutionary systems-theoretical account of levels based on building blocks seems to provide a promising framework for developing a globally and universally applicable hierarchy of levels of material composition. The concept of building blocks is insofar relevant to the development of a domain granularity framework for the life sciences, as I argue that it gives rise to a compositional granular perspective of building blocks that represents the abovementioned ideal bona fide spatio-structural granular perspective that functions as organizational backbone for the granularity framework.

I characterize a building block as follows:

- A building block possesses a *physical covering* that is comparable to what Jagers op Akkerhuis and Van Straalen [18] have referred to as an *interface*. The physical covering not only demarcates the building block from its environment, making it a *spatio-structurally* bona fide *entity*, but also functions as a *physical barrier* that protects a specific *inside milieu* from the *outside milieu* that surrounds the building block, establishing a micro-ecosystem within the building block that follows different functional vectors than the outside macro-ecosystem. The physical covering relates also to Smith et al.’s [71] account of causal unity via physical covering (see above). It is, however, on the one hand more general, because it treats also electron shells as a physical covering (see below), and on the other hand more specific, because it includes also functional aspects of the physical covering. Moreover, contrary to the mathematical account of boundary followed by Smith et al. [71, 113–116], the physical covering of a building block is itself a three-dimensional material entity and is therefore rather a *boundary region* [98]. This is an important aspect, as it provides building blocks with what Wimsatt called *robustness* (“Things are robust if they are accessible (detectable, measurable, derivable, definable, producible, or the like) in a variety of independent ways”, [117], p. 210f; see also [118]). The physical covering not only determines the boundary region of a building block, but is itself a bona fide functional unit that not only provides the surface of the boundary of the building block, but also bears the dispositions with which the building block interacts and communicates with its environment.
- A building block is not only a spatio-structurally bona fide entity, but also a bona fide *functional unit* that possesses its own regulatory machinery with feedback mechanisms, so that to a certain degree it is self-organized and self-maintained. Building blocks represent localized islands of order that have a *stable internal organization* and maintain their integrity during typical interactions. A building block usually lives/exists longer than its constituent parts and its behavior is predictable for the situations typically found in its environment.
- New types of building blocks come into being as a result of (cosmic) evolution.
- A building block is able to interact with other building blocks to form aggregates and more complex building blocks (Simon’s *assemblies* [29]). Building blocks of a coarser level are composed of building blocks of finer level(s). As a consequence, a building block of a coarser level is necessarily *existentially dependent* on a building block of some finer level, resulting in a *hierarchy of irreducible levels*. Building blocks of coarser levels can only evolve after finer level building blocks have evolved.

Building blocks thus provide nature with its universal inventory of matter, just like lego-bricks with which increasingly complex structures can be built. The evolution of a new type of building block that constitutes a new and coarser level always corresponds with a substantial increase in material diversity and adds a new dimension to the spatio-structural space for evolution to explore. Building blocks are spatio-structurally, functionally, developmentally and evolutionarily both integrated and stable, but at the same time increase nature’s overall evolvability.

#### Non-biological building blocks

According to the characterization above, the *electron shell* is a unit of physical covering of a building block (cf. [18]). There are two types of material entities that are covered by electron shells: atoms and molecules. In an *atom*, a cloud of electron ‘waves’ surrounds the nucleus. It physically covers the atom and also determines the interaction of the atom with the entities of its environment. Electromagnetically, one can clearly identify a stable inside milieu that is protected from an outside milieu via the electron shell.

Electron shells from several atoms can bind to form a *molecule*. In a molecule, several atoms share a common electron shell, forming the building blocks of the next coarser level of granularity. This also applies to lumps of metal, in which several atomic nuclei share a common electron shell. In metals, however, the sharing of electrons is not localized between two atoms (i.e., covalent bond), but instead *free* electrons are shared among a lattice of positively charged ions (i.e., metallic bonding). Therefore, causal unity via physical covering in the here proposed concept of building blocks would include atoms, lumps of metal and molecules as bona fide *objects* in the sense of Smith et al. [71] (for the sake of simplicity, from here on I include metals in molecules and also treat ionic compounds as molecules; in other words, I include all compositions of atoms in molecules that are based on intramolecular forces).

Molecules can further combine to form bona fide objects based on intermolecular forces such as a portion of water that consists of several water molecules that become aggregated due to hydrogen bonds. These objects, however, do not constitute building blocks themselves, because they lack a common physical covering. Instead, they are bona fide *aggregates of molecule building blocks*.

#### Biological building blocks delimited by a plasma membrane

Biological building blocks are building blocks that are biological material entities that can be found universally across a wide range of taxonomic groups. Their prototypical forms have evolved during biological evolution and have been very successful in combining and recombining finer level building blocks to built building blocks of the next coarser level. Because biological building blocks continue to evolve, a variety of different forms exist, all of which, however, share some common characteristics so that they can be referred to as instances of the same set of prototypical building block categories. As a consequence, biological building blocks can considerably vary in size, in particular across different taxa. Correlating biological building block levels with scale levels across different taxa is therefore often impossible.

In order to identify a biological building block, we must identify, which types of biological physical coverings meet the criteria discussed above for physical covering of a building block. The *biological plasma membrane* qualifies as such a physical covering. Various biological material entities are surrounded and naturally demarcated by a biological plasma membrane, with its most important component being amphipathic molecules. Amphipathic molecules such as phospholipids possess both a hydrophobic and a hydrophilic region. According to the fluid mosaic model, the membrane is a fluid structure that is arranged in a mosaic-like fashion with different kinds of proteins embedded in or attached to a phospholipid bilayer [27]. This supramolecular structure is thus an *aggregate of molecules* that is primarily held together by hydrophobic interactions, which are significantly weaker than covalent bonds, but nevertheless strong enough to maintain its structural integrity. Therefore, following Smith et al.’s [71] definition of bona fide objects, each bio-membrane is a bona fide object that is a molecule aggregate that is causally unified via internal physical forces, i.e., hydrophobic interactions.

A specific degree of fluidity is essential for the proper functioning of the membrane as a semi-permeable barrier and for its embedded enzymatic proteins, many of which require being able to move within the membrane for their activity [27]. Whereas the phospholipids provide the spatio-structural skeleton of the membrane, its various types of proteins determine most of its functions, ranging from selective transport across the membrane, to various enzymatic activities, signal transduction, cell-cell recognition, intercellular joining such as gap junctions or tight junctions, and attachment to the cytoskeleton and the ECM. Each type of plasma membrane can be characterized by its set of membrane proteins.

There are two types of biological material entities that are covered by plasma membranes: *cells* (prokaryotic as well as eukaryotic cells) and *organelles*, the latter of which are membrane-enclosed structures within eukaryotic cells, including nucleus, endoplasmatic reticulum, lysosome, mitochondrion, peroxisome, cisternae of the Golgi apparatus, central vacuole, chloroplast, and all vesicles and vacuoles. In the here suggested strict sense of *organelle* as a membrane-enclosed material entity within eukaryotic cells, the Golgi apparatus itself is not an organelle, but an aggregate of organelles, because its cisternae are physically disconnected organelles themselves.

Cells and organelles are thus biological building blocks and therefore spatio-structural as well as functional bona fide entities. When only considering the topology of the membranes, one must, however, distinguish a building block *‘single-membrane-enclosed entity’* that comprises all organelles and prokaryotic cells, from a building block *‘membrane-within-membrane entity’* that comprises eukaryotic cells, which are membrane-enclosed entities that have membrane-enclosed entities as their parts.

Several eukaryotic cells can fuse to form a syncytium, which is a multinucleated cell (e.g., skeletal muscles and cardiac muscle in humans and the syncytiotrophoblast in vertebrates, which is the epithelial covering of a placenta), or they can conduct multiple nuclear divisions without accompanying cytokinesis to form coenocytes. In both cases several nuclei share the same cell membrane, thus, forming mutliplets of eukaryotic cells. However, although topologically substantially different to eukaryotic cells with a single nucleus, syncytia and coenocytes are nevertheless membrane-within-membrane entities.

Prokaryotic cells as well as eukaryotic cells can become aggregated such as can be seen in bacterial colonies or in epithelia of multi-cellular animals, forming bona fide objects in the sense of Smith et al. [71] based on causal unity via internal physical forces. These objects, however, do not constitute building blocks themselves, because they lack a common physical covering. Instead, they are bona fide *aggregates of molecule and cell building blocks*.

#### Biological building blocks delimited by an epithelium

An epithelium is another type of biological physical covering that qualifies as a covering of a building block. An *epithelium* is composed of polarized cells that form a tightly packed continuous single-layered sheet of cells. Every epithelium has an apical surface and a lower basal surface, the latter of which is attached to a basal lamina that is a layer of ECM secreted by the epithelial cells. The basal lamina acts as a filter for any molecules attempting to pass into the space covered by the epithelium. Many epithelial cells possess microvilli at their apical side, increasing the surface area of this side of the epithelium, which is important for functions of secretion, absorption, and sensory functions. The apical side can also possess a motile cilium for pushing substances along the apical surface of the epithelium. Tight junctions in case of vertebrates and septate junctions in case of invertebrates connect the plasma membranes of adjacent epithelial cells through specific proteins in the membranes, forming a continuous semi-permeable seal around the epithelial cells that prevents fluids from moving through the intercellular spaces of the epithelial cells and thus across the epithelium. According to Smith et al.’s [71] definition of bona fide objects, each epithelium as such is thus a cell aggregate that forms a bona fide object that is causally unified via internal physical forces, i.e., tight junctions or septate junctions respectively. The epithelium functions as a diffusion barrier. The epithelium lining the blood vessels of Tetrapoda, for example, functions as a hemato-encephalic barrier that prevents some substances in the blood (e.g., some toxins and pathogens) to come in contact with brain tissue. This protects a specific inside milieu within the brain from its outside milieu. Epithelia can have various additional functions, ranging from selective absorption of water and nutrients, protection, elimination of waste products, secretion of enzymes and hormones, transcellular transport, to sensory functions. All animal glands, for instance, are made of epithelial cells.

There are two types of anatomical entities that are covered by epithelia: *organisms with an epidermis*, and *epithelially-delimited compartments*, the latter of which are epithelium-enclosed structures within multi-cellular animals, including, for instance, the circulatory system in humans, lungs in vertebrates, and the intestine in animals. Therefore, *‘epithelially-delimited compartment’* and *‘epithelially-delimited multi-cellular organism’* are both biological building blocks, the latter of which are epithelium-within-epithelium entities.

Epithelially-delimited compartments can aggregate such as the digestive system in humans, which consists of the gastrointestinal tract together with all accessory organs of digestion (tongue, salivary glands, pancreas, liver, and gallbladder). Although one can argue that such an aggregate forms a functional bona fide unit, it does not constitute a building block, because it lacks a common physical covering. Instead, it is an *aggregate of molecules, cells and epithelially-delimited compartment building blocks* (see discussion below).

### Results I: Spatio-structural granular perspectives

#### Compositional building block (CBB) granular perspective

On the basis of the abovementioned characterization of building blocks one can identify the following prototypical building blocks: ‘atom’ < ‘molecule’ (including metals and ionic compounds) < ‘single-membrane-enclosed entity’ (i.e., most organelles and all prokaryotic cells) < ‘membrane-within-membrane entity’ (i.e., eukaryotic cell) < ‘epithelially-delimited compartment’ (i.e., some, but not all of the entities that are commonly referred to as organs) < ‘epithelially-delimited multi-cellular organism’ (i.e., organisms with an epidermis).

Comparable to the hierarchy proposed by Jagers op Akkerhuis and Van Straalen [18], the resulting hierarchy of levels of building blocks ranks complexity solely in a strict layer-by-layer fashion—it is a robust hierarchy that does not allow for bypasses, such as the sequence ‘sand’ < ‘stone’ < ‘planet’ allows bypassing the ‘stone’ level by constructing a planet from sand alone [18]. Levels in an aggregate hierarchy on the other hand allow such bypassing (see also distinction of aggregates and levels of organization in [35]). The hierarchy of levels of building blocks provides what Craver [23] would call *monolithic levels* that reach across all material domains of reality and that are *globally and universally applicable.* Because the concept of a building block is based on an evolutionary interpretation, it explicitly predicts the diversification of newly evolved building blocks of a given level, with each higher level exhibiting the possibility of an exponentially larger number of different types of entities associated with a building block to be evolved—the number of possible types of molecules is exponentially larger than the number of possible types of atoms. When considering that actual material entities can be composed of a multiplicity of different possible combinations (i.e., aggregates) of those building blocks, comparable to constructions made from lego-bricks, the diversity of possible types of material entities increases even more with each newly evolved building block.

**Fig. 4:**
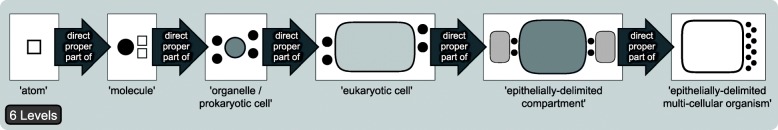
Compositional Building Block (CBB) Granular Perspective. The different building blocks are granulated according to the direct proper parthood granulation relation (the large dark arrows). The granulation is of the non-scale dependent single-relation-type granularity type (*nrG* [61]), and uses the combination of the granulation relation together with the common properties of all categories of the building block type as its granulation criterion. Due to the cumulative constitutive organization, finer-level building block entities can be considered to be parts associated with coarser-level building block entities, for instance, ECM being an associated part of a eukaryotic cell

On the basis of this concept of building blocks and the implicit hierarchy of building blocks, a granular perspective of levels of building blocks can be characterized using Keet’s general formal theory of granularity [61]. The *subject domain* in all granularity perspectives discussed in the following is restricted to cumulative-constitutively organized material entities. The bona fide partition of a given building block entity into its building block components represents a qualitative compositional partition (as opposed to a qualitative regional partition or a quantitative resolution-based partition). This *compositional building block (CBB) granular perspective* is based on a *direct proper parthood* relation between instances of different top-level categories of building blocks (see discussion below), and thus has the *granulation criterion* (Fig. 4):

**Table:** 

building block	directProperPartOf	building block;
building block	hasDirectProperPart	building block*.*

According to Keet’s formal theory of granularity, this perspective has a granulation of the non-scale dependent single-relation-type *granularity type* (*nrG* [61]; also called non-scale dependent primitive granularity type, *npG* [60]). It is based on the direct proper parthood relation as its *granulation relation*. Entities residing in adjacent CBB granularity levels are thus related through the direct proper parthood relation. In order to constitute a CBB granular perspective, instances of at least two different categories of building block must exist, of which instances of one category are direct proper parts of instances of the other. In other words, the levels of the CBB granular perspective are demarcated from one another according to the properties of the top-level categories of building block and they are ordered from finest to coarsest granularity level according to the direct proper parthood relation. The number of levels within the CBB granular perspective directly depends on the number of top level categories of building blocks identified (Fig. 4).

According to the underlying cumulative constitutive organization, for all instances of building block holds (*compositional object granularity perspective* [58]):

1. An instance of a building block is not necessarily a proper part of an instance of some building block of the adjacent coarser CBB granularity level.
2. Every instance of a building block, except for those belonging to the finest CBB granularity level, has at least two instances of building blocks of finer levels as its proper parts.
3. The instance of the building block that is granulated is the maximum entity that belongs to the coarsest CBB granularity level, and every other instance of a building block belonging to this granulation is a proper part of this maximum entity. However, because this maximum entity is cumulative-constitutively organized, its direct proper parts not necessarily all belong to the second coarsest CBB granularity level.

Because each entity belonging to a specific CBB granularity level represents a BFO ‘object’, we can distinguish six different spatio-structural frames of reference, which ***can be ordered according to the associate CBB granularity levels from finer to coarser spatio-structural frames of reference***: an atom, a molecule, an organelle/prokaryotic cell, a eukaryotic cell, an epithelially-delimited compartment and an epithelially-delimited multi-cellular organism frame of reference. Each such spatio-structural frame of reference has its own set of granular perspectives. As a consequence, whereas any given material entity can belong to six different spatio-structural granular perspectives, it can belong to maximally one CBB granularity level.

Moreover, because a building block is defined as a bona fide spatio-structural entity as well as a bona fide functional unit, the CBB granular perspective comes close to the ideal organizational backbone for the development of a domain granularity framework for the life sciences. Conceptually, it therefore takes in a central position within this framework.

#### Compositional building block cluster (CBB-C) granular perspectives

**Fig. 5:**
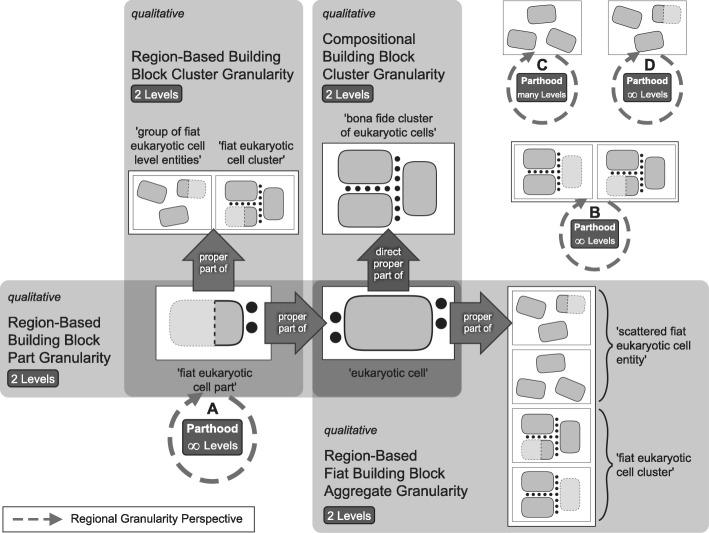
Set of Granular Perspectives within a given spatio-structural Frame of Reference. The figure shows all qualitative granular perspectives that the domain granularity framework for the life sciences distinguishes for any given spatio-structural frame of reference and thus any corresponding CBB granularity level (here, the set of perspectives for the eukaryotic cell level as an example). The large dark arrows indicate the granulation relation and the white boxes contain the granulated entity types. **a** = Region-Based Fiat Building Block Part Granularity Perspective; **b** = Region-Based Fiat Building Block Cluster Granularity Perspective; **c** = Region-Based Group of Building Block Level Objects Granularity Perspective; **d** = Region-Based Group of Fiat Building Block Level Entities Granularity Perspective (see also Table 1)

As already mentioned above, building blocks can aggregate to form bona fide entities that are not building blocks themselves. Each spatio-structural frame of reference (i.e., atomic, molecular, single-membrane-enclosed, membrane-within-membrane, etc.) accommodates two distinct categories of bona fide entities. The eukaryotic frame of reference, for instance, includes ‘eukaryotic cell’ as well as ‘bona fide cluster of eukaryotic cells’. Whereas the former is a building block and thus belongs to the respective granularity level of the CBB granular perspective, the latter is not, because only the former is based on the more restrictive causal unity via physical covering as criterion for their bona fideness. The bona fideness of ‘bona fide cluster of eukaryotic cells’, in contrast, is only based on the more general causal unity via internal physical forces. However, because they represent aggregates of building blocks that can be partitioned into their component object parts that belong to the same spatio-structural frame of reference, one can characterize the corresponding qualitative compositional partitions as *compositional building block cluster (CBB-C) granular perspectives* (see Fig. 5). Each CBB granularity level has its own corresponding CBB-C granular perspective. This CBB-C granular perspective is based on a *direct proper parthood* relation between instances of building blocks of a given spatio-structural frame of reference and their corresponding bona fide clusters, and thus has the building-block-level-specific *granulation criterion* (Fig. 5):

**Table:** 

‘building block’ ^X^	directProperPartOf	‘bona fide cluster of [building block]s’ ^X^;
‘bona fide cluster of [building block]s’ ^X^	hasDirectProperPart	‘building block’ ^X^;

X = a specific spatio-structural frame of reference.

Like the CBB granular perspective, the CBB-C perspective has a granulation of the non-scale dependent single-relation-type *granularity type* (*nrG* [61]) and is based on the direct proper parthood relation as its *granulation relation*. Because the domain and range of the granulation relation differ according to the granulation criterion, the granulation relation is *not* transitive and thus each of the CBB-C perspectives includes only two distinct granularity levels.

#### Region-based granular perspectives

Besides the two types of compositional granular perspectives, each spatio-structural frame of reference has its own set of seven different associated region-based granular perspectives (for an overview, see Fig. 5). The different perspectives, together with their specific granulation criterion, granulation type, and granulation relation are listed in Table 1. They differ only with respect to their granulation type, but they all share the same non-scale dependent single-relation-type *granularity type* (*nrG* [61]) and are all based on the *proper parthood* relation as their *granulation relation*.

**Table 1:** List of Region-Based Granularity Perspectives for each given spatio-structural frame of reference (compare with Fig. 5); *nrG* = non-scale dependent single-relation granularity type, sgrG = scale-dependent grain size with respect to resolution [61]

Level-Specific Granularity Perspective	Granulation Criterion			Granularity Type	Granulation Relation	# Levels
Region-Based Building Block Cluster Granularity Perspective	‘fiat [building block] part’ ‘fiat [building block] part’ ‘group of fiat [building block] level entities’ ‘fiat [building block]cluster’	properPartOf properPartOf hasProperPart hasProperPart	‘group of fiat [building block] level entities’ OR ‘fiat [building block] cluster’; ‘fiat [building block] part’ OR ‘fiat [building block] part’	*nrG*	*proper parthood*	2
Region-Based Building Block Part Granularity Perspective	‘fiat [building block] part’ ‘[building block]’	properPartOf hasProperPart	‘[building block]’; ‘fiat [building block] part’	*nrG*	*proper parthood*	2
Region-Based Fiat Building Block Aggregate Granularity Perspective	‘[building block]’ ‘[building block]’ ‘fiat [building block] cluster’ ‘scattered fiat [building block] entity’	properPartOf properPartOf hasProperPart hasProperPart	‘fiat [building block] cluster’ OR ‘scattered fiat [building block] entity’; ‘[building block]’ OR ‘[building block]’	*nrG*	*proper parthood*	2
Region-Based Fiat Building Block Part Granularity Perspective	‘fiat [building block] part’ ‘fiat [building block] part’	properPartOf hasProperPart	‘fiat [building block] part’; ‘fiat [building block] part’	*nrG*	*proper parthood*	∞
Region-Based Fiat Building Block Cluster Granularity Perspective	‘fiat [building block] cluster’ ‘fiat [building block] cluster’	properPartOf hasProperPart	‘fiat [building block] cluster’; ‘fiat [building block] cluster’	*nrG*	*proper parthood*	∞
Region-Based Group of Building Block Level Objects Granularity Perspective	‘group of [building block] level objects’ ‘group of [building block] level objects’	properPartOf hasProperPart	‘group of [building block] level objects’; ‘group of [building block] level objects’	*nrG*	*proper parthood*	many
Region-Based Group of Fiat Building Block Level Entities Granularity Perspective	‘group of fiat [building block] level entities’ ‘group of fiat [building block] level entities’	properPartOf hasProperPart	‘group of fiat [building block] level entities’; ‘group of fiat [building block] level entities’	*nrG*	*proper parthood*	∞

These seven general types of region-based granular perspectives result in a set of 49 different specific region-based granular perspectives within the domain granularity framework for the life sciences. This set is sufficient to model all possible region-based partition relations between any given pair of spatio-structural entities for a given spatio-structural frame of reference.

#### Function-based and history/evolution-based granular perspectives

In analogy to the distinction between the CBB and the region-based granular perspectives for spatio-structural entities, one can also distinguish between a *compositional functional unit (CFU) granular perspective* (which corresponds with the mechanism-based approach to levels [119–123]) and various *region-based functional entity granular perspectives*, as well as between a *compositional historical/evolutionary unit (CH/EU) granular perspective* and various *region-based historical/evolutionary entity granular perspectives* respectively.

The partition of a given functional unit or historical/evolutionary unit into components that themselves are functional units or historical/evolutionary units represents a qualitative compositional partition. The functional compositional partition is based on a *direct proper functional parthood* relation (which can be derived from the direct proper parthood relation by restricting its domain and range to instances of ‘functional unit’) between instances of different sub-categories of ‘functional unit’ (see next chapter), which thus represents the *granulation relation* of the CFU granular perspective. Its *granulation criterion* is:

The historical/evolutionary compositional partition, on the other hand, is based on a *direct proper historical/evolutionary* (DirPropHistEvol) parthood relation (which can be derived from the direct proper parthood relation by restricting its domain and range to instances of ‘historical/evolutionary unit’) between instances of different sub-categories of ‘historical/evolutionary unit’ (see next chapter), which thus represents the *granulation relation* of the CH/EU granular perspective. Its *granulation criterion* is:

According to Keet’s formal theory of granularity, both perspectives have a granulation of the non-scale dependent single-relation-type *granularity type* (*nrG* [61]). Contrary to the CBB granular perspective, however, an underlying hierarchy of levels of functional or historical/evolutionary building blocks that defines the number of possible levels of a CFU or CH/EU granular perspective, like the CBB granular perspective does for spatio-structural entities, is missing. Neither the CFU nor the CH/EU granular perspective can be based on a hierarchy of monolithic levels of functional or historical/evolutionary units that are globally and universally applicable and reach across all domains of the life sciences—to stay within the metaphor: we do not know reality’s inventory of functional and historical/evolutionary lego-bricks. Instead, representatives of different species, even different particular biological material entities, can substantially differ in the number and structure of their CFU and CH/EU granular perspectives.

Because we do not distinguish between different sub-types of functional and historical/evolutionary causal unity, like we do with causal unity via internal physical forces and via physical covering for spatio-structural entities, there is no analog for the CBB-C granular perspective for functional and historical/evolutionary entities. However, one can differentiate various region-based functional and region-based historical/evolutionary granular perspectives in analogy to the various region-based granular perspectives for spatio-structural entities, which I do not discuss here for lack of space.

**Table:** 

‘functional unit’	directProperFunctionalPartOf	‘functional unit’;
‘functional unit’	hasDirectProperFunctionalPart	‘functional unit’.

**Table:** 

‘hist/evol unit’	DirPropHistEvolPartOf	‘hist/evol unit’;
‘hist/evol unit’	hasDirPropHistEvolPart	‘hist/evol unit’.

### 2nd step: Dealing with specific problems resulting from the cumulative constitutive Organization of Reality

#### Extending and rearranging BFO’s top-level category of ‘material entity’ to accommodate different frames of reference

**Fig. 6:**
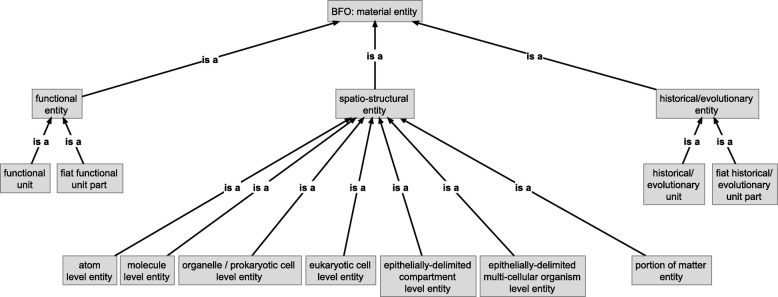
Top-Level Subclasses of ‘material entity’ and ‘spatio-structural entity’. The labeled grey boxes represent classes. The class ‘spatio-structural entity’ is characterized in reference to causal unity via internal physical forces, ‘functional entity’ in reference to causal unity via bearing a specific function, and ‘historical/evolutionary entity’ in reference to causal unity via common historical/evolutionary origin. As a consequence of the perspective-dependence of bona fideness, these three classes are not disjoint. The functional and historical/evolutionary entities are further differentiated according to disjoint categories of bona fide units and fiat unit parts. Spatio-structural entities are further differentiated in correspondence with the granularity levels of the compositional building block granular perspective (see discussion in text), ranging from ‘atom level entity’ to ‘epithelially-delimited multi-cellular organism level entity’, but include not only the respective bona fide entities of that level, but also their corresponding object aggregate and fiat object part entities. Because bona fideness is not only perspective-dependent, but also granularity-dependent, each building block level has its own spatio-structural frame of reference and thus its own perspective. Due to the cumulative-constitutive organization of biological entities, entities from finer spatio-structural frames of reference (e.g., molecules) must be represented in coarser frames of reference (e.g., eukaryotic cell) as fiat portions of matter. These representations are covered through the ‘portion of matter entity’ class (see also Fig. 8)

The frame-dependence of the relevance of different types of causal unity and the resulting differentiation of three basic categories of granular perspectives and their corresponding basic frames of reference (i.e., spatio-structural, functional, historical/evolutionary), together with the differentiation of spatio-structural frames of reference in dependence on the number of granularity levels identified for the CBB granular perspective (i.e., atomic, molecular, etc.), reflect a basic distinction of sub-categories of ‘material entity’. I therefore suggest the following top-level classes for BFO’s ‘material entity’ (see Fig. 6). The classes ‘functional entity’, ‘historical/evolutionary entity’, and ‘spatio-structural entity’ distinguish foundational types of material entity based on their underlying type of causal unity, which is causal unity via bearing a specific function, causal unity via common historical/evolutionary origin, and causal unity via internal physical forces, respectively. And because causal unity via physical covering supervenes on causal unity via internal physical forces, the latter covers the former [98]. Because of the frame-dependence of the relevance of these different types of causal unity, these three classes are *not disjoint*. As a consequence, some given material entity may instantiate ‘functional entity’, ‘historical/evolutionary entity’, and ‘spatio-structural entity’ at the same time.

On the basis of the identification of different spatio-structural frames of reference, I can now suggest the following top-level classes for ‘spatio-structural entity’: ‘atom level entity’, ‘molecule level entity’, ‘organelle/prokaryotic cell level entity’, ‘eukaryotic cell level entity’, ‘epithelially-delimited compartment level entity’, ‘epithelially-delimited multi-cellular organism level entity’ (see Fig. 6). *Each of these categories corresponds with one of the spatio-structural frames of reference.* Due to the frame-dependence, these six classes of ‘spatio-structural entity’ are also *not disjoint*, because some given spatio-structural entity may be a molecule, but at the same time also a fiat organelle part and a fiat eukaryotic cell part.

On the basis of (i) the identification of different spatio-structural frames of reference, (ii) the implications of a cumulative constitutive organization of biological material entities, and (iii) because bona fideness is granularity- and thus frame-dependent [77, 98], I treat all bona fide and fiat entities from a given spatio-structural frame of reference in coarser frames of reference as *fiat entities*. As a consequence, ‘portion of matter entity’ is introduced as another top level class of ‘spatio-structural entity’ in addition to the set of building-block-level-specific classes. It refers to the *representation* of entities from a finer spatio-structural frame of reference level at coarser frame-levels (see next chapter and Figs. 6 and 8).

Regarding the functional and historical/evolutionary entities, one can only distinguish bona fide and fiat entities with respect to their corresponding frames of reference. Therefore, ‘functional entity’ has the top-level classes ‘functional unit’, which comprises all bona fide functional entities, and ‘fiat functional unit part’, which comprises all fiat functional entities respectively. Accordingly, one can distinguish ‘historical/evolutionary unit’ from ‘fiat historical/evolutionary unit part’. Because for functional and historical/evolutionary entities no backbone granularity scheme exists that is comparable to the building block levels hierarchy and the associated CBB granular perspective discussed above, no additional differentiation into further subclasses is suggested. One could, of course, differentiate functional entities based on the type of functions they bear and thus the type of corresponding processes (i.e., functionings), into functional units of locomotion, physiology, ecology, development, and of reproduction and propagation, and historical/evolutionary entities into historical units of development, heredity, and of evolution and developmental, genealogical and evolutionary lineages [77].

**Fig. 7:**
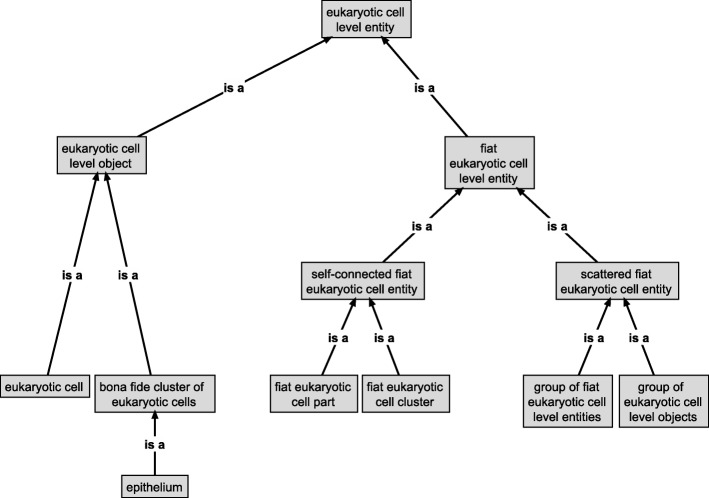
Top-Level Subclasses of ‘eukaryotic cell level entity’. Eukaryotic cell level entities are differentiated into a bona fide ‘eukaryotic cell level object’ and a ‘fiat eukaryotic cell level entity’ class, which are disjoint. The former is differentiated based on its underlying type of causal unity into ‘eukaryotic cell’, which is based on physical covering, and ‘bona fide cluster of eukaryotic cells’, which is based only on internal physical forces and not on physical covering. The fiat eukaryotic cell level entities are differentiated based on their self-connectedness into the disjoint subclasses ‘self-connected fiat eukaryotic cell entity’ and ‘scattered fiat eukaryotic cell entity’. See text for more details

Because each spatio-structural frame of reference includes not only the corresponding building block and its bona fide aggregates, but also their corresponding fiat building block parts and fiat building block aggregates, each direct subclass of ‘spatio-structural entity’ includes all corresponding fiat and bona fide entities. In other words, I interpret BFO’s categories ‘object’, ‘object aggregate’, ‘fiat object part’ as being applicable to each spatio-structural frame of reference. Therefore, I consider the distinction between fiat and bona fide material entities to be foundational for each spatio-structural frame of reference. Taking the ‘eukaryotic cell level entity’ (i.e., membrane-within-membrane frame of reference) as an example, this approach results in the basic distinction of ‘eukaryotic cell level object’ and ‘fiat eukaryotic cell level entity’ (see Fig. 7).

The ‘eukaryotic cell level object’ corresponds with BFO’s ‘object’ category. Depending on which type of causal unity is relevant for the given object entity, I distinguish two types of objects for each spatio-structural frame of reference and thus two subclasses for each direct subclass of ‘spatio-structural entity’. On the one hand the entities that belong to the corresponding CBB granularity level, which are objects that are based on the more specific causal unity via physical covering. In the case of ‘eukaryotic cell level object’ this would be ‘eukaryotic cell’ (see Fig. 7), or ‘molecule’ in the case of ‘molecule level object’. On the other hand, because building blocks can aggregate to form bona fide clusters based on the more general causal unity via internal physical forces, another object category is required to deal with these types of objects. Thus, ‘eukaryotic cell level object’ would not only have ‘eukaryotic cell’ as its direct subclass, but also ‘bona fide cluster of eukaryotic cells’, for example, those cells that together build an epithelium (which provides the physical covering of the building block entities of the next coarser spatio-structural frame of reference). Or, in case of ‘molecule level object’, ‘bona fide cluster of molecules’ can form a bio-membrane or a chitin cuticula, both of which are bona fide objects that are based on causal unity via internal physical forces (as opposed to the building block itself, which is additionally based on causal unity via physical covering).

These building block level objects are contrasted with fiat building block level entities, which cover BFO’s ‘fiat object part’ and ‘object aggregate’ and comprise all material entities that possess spatio-structurally no causal unity (neither via internal physical forces nor via physical covering—note that this fiatness depends on the granularity level of the building block entity, which provides the relevant spatio-structural frame of reference in this context).

Fiat building block entities can be further differentiated based on whether they are spatio-structurally self-connected, giving rise to two distinct subclasses. In case of ‘fiat eukaryotic cell level entity’ this results in the distinction of ‘self-connected fiat eukaryotic cell entity’ and ‘scattered fiat eukaryotic cell entity’ (Fig. 7). Self-connected fiat entities can be further differentiated into fiat building block parts and thus the building block level specific correlate to BFO’s ‘fiat object part’, and fiat building block clusters. For the eukaryotic cell level, the former would translate into ‘fiat eukaryotic cell part’ and the latter into ‘fiat eukaryotic cell cluster’, respectively. A scattered fiat entity, on the other hand, can be further differentiated based on the type of its scattered component parts. If all scattered component parts are building block level objects that correspond to the relevant spatio-structural frame of reference, the scattered entity is a group of building block level objects (e.g., ‘group of eukaryotic cell level objects’). However, if at least one of its component parts is a fiat building block level entity, the scattered entity is a group of building block level entities (e.g., ‘group of fiat eukaryotic cell level entities’) (see Fig. 7). For a distinction of (i) *groups* based on *metric proximity* as the relation between its parts versus (ii) *clusters* based on *topological adherence* as the relation between its parts see Vogt et al. [87, 124].

#### Consequence from the cumulative constitutive organization of biological material entities and the frame-dependence of their representation

The abovementioned direct subclasses of ‘spatio-structural entity’ must accommodate all types of material entities found in cumulative-constitutively organized biological material entities. Therefore, its sub-classes always refer to the building block entity of the corresponding spatio-structural frame of reference, independent of whether finer-level entities are also involved. In other words, ‘eukaryotic cell’ or ‘fiat eukaryotic cell part’ comprise all types of eukaryotic cell or eukaryotic cell part entities, with and without associated portions of connected ECM, and ‘epithelially-delimited compartment’ comprises all types of epithelially-delimited compartments, with and without associated portions of connected molecular matter and portions of connected tissue (see also Figs. 4 and 5). Therefore, when we talk about a eukaryotic cell cluster, this can refer to a cluster of cells with surrounding ECM, but it could also refer to a cluster of cells without surrounding ECM. This is a rather pragmatic choice, as the alternative would require distinguishing various categories to cover each possible combination of different levels of building block entities that can be found in a cumulative constitutive organization, which would result in a tremendous increase in top-level classes [87, 124]. This would neither be convenient and intuitive to use, nor really necessary.

**Fig. 8:**
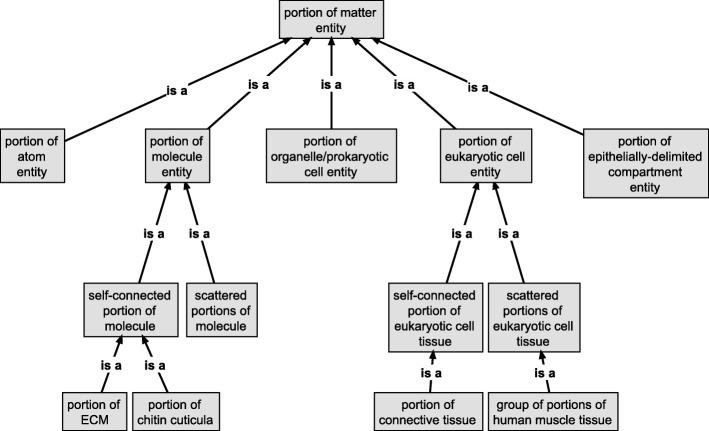
Top-Level Subclasses of ‘portion of matter entity’. The entities of each building block level, except for the coarsest level of epithelially-delimited multi-cellular organisms, can be represented as a respective portion of matter entity in coarser spatio-structural frames of reference. Therefore, ‘portion of matter entity’ is differentiated into building block level specific subclasses. Further differentiations are shown for the classes ‘portion of molecule entity’ and ‘portion of eukaryotic cell entity’, which are based on whether the entity is a self-connected portion of matter, for instance, a portion of ECM or a portion of connective tissue, or a group of scattered portions, for instance, the group of portions of muscle tissues in a human being

Because biological material entities are usually cumulative-constitutively organized (see discussion above), entities of finer building block levels can exist outside of building blocks of coarser levels, for instance, molecules outside of eukaryotic cells. Unfortunately, these finer level entities cannot be covered with the categories of the coarser levels, since they are neither bona fide objects nor fiat object parts entities of this object level—a molecule that exists outside of eukaryotic cells does neither represent a eukaryotic cell level object nor a fiat eukaryotic cell level entity. In other words, the adequate classes for referring to these entities belong to a different and finer spatio-structural frame of reference. However, respective entities still must be represented in the frame of reference of the coarser level (see *sortation-by-type* and *type granularity trees* problematic discussed in chapter *Biological Reality: The Problem with the Cumulative Constitutive Hierarchy*, see Fig. 2). As already mentioned above, I therefore introduce the class ‘portion of matter entity’. For instance, eukaryotic cell clusters and single eukaryotic cells, as well as molecule clusters and single molecules, can exist outside of epithelially-delimited compartments (see also Fig. 2). However, none of the subclasses of ‘epithelially-delimited compartment level entity’ can accommodate these material entities. They therefore must be covered by the classes ‘portion of molecule entity’ and ‘portion of eukaryotic cell entity’ respectively, which are frame-of-reference-specific subclasses of ‘portion of matter entity’ (see Figs. 6 and 8).

A *portion of matter* is a non-countable entity (c.f. masses [125]; amount of matter [126]; portion of unstructured stuff [127]; see also body substance [64]; and portion of body substance [56]). In order to count the number of component parts of a portion of matter, one would have to change the spatio-structural frame of reference from the current frame to a frame of a finer level that corresponds with the component parts of that portion. Thus, a cluster of molecules, for instance, the chitin cuticula that forms the exoskeleton in insects, which is a bona fide cluster of chitin molecules and thus instantiates ‘molecule level object’ at the molecular frame of reference, is represented as a self-connected (fiat) portion of molecular matter at all coarser spatio-structural frames of reference. The individual molecules that build the cluster cannot be individually differentiated anymore at reference levels coarser than the molecular level, because their bona fideness disintegrates at these coarser levels [87], which is why all portions of matter are treated as fiat entities. If a portion of matter consists of a mixture of building block entities of different spatio-structural frames of reference such as a portion of connective tissue that is a group of cells embedded in a cluster of collagen molecules, the coarsest building block entity is used for classifying it, which in this case would be a portion of connective tissue. Portions of tissue always refer to cell aggregates. Most cells in multi-cellular organisms are surrounded by a complex cluster of molecules, i.e., the ECM.

Because entities belonging to a finer spatio-structural frame of reference are always represented as non-countable fiat portions of matter in coarser spatio-structural frames of reference, one can only distinguish between self-connected and scattered portions. In case of ‘portion of eukaryotic cell entity’, one can thus distinguish ‘self-connected portion of eukaryotic cell tissue’ from ‘scattered portions of eukaryotic cell tissue’ respectively (see Fig. 8).

#### Cross-granular multiple instantiation

Due to its granular nature, any given biological material entity always instantiates several different material entity categories at the same time, one for each spatio-structural frame of reference [87]. For example, every instance of ‘eukaryotic cell’ instantiates at finer frames of reference also ‘bona fide cluster of molecules’ and ‘bona fide cluster of atoms’, because a eukaryotic cell is a bona fide composition of clustered molecules and at the same time also a bona fide composition of clustered atoms. At coarser frames of reference it also instantiates frame-specific classes. Which class is instantiated at those coarser frames, however, depends on the particular eukaryotic cell. If it exists outside of any epithelially-delimited compartment, it is not covered by any level-specific subcategory of ‘epithelially-delimited compartment entity’ and therefore instantiates some category of ‘portion of eukaryotic cell entity’ (see discussion in previous chapter). If it is part of an epithelially-delimited compartment it instantiates ‘fiat epithelially-delimited compartment part’.

One could, of course, define a class ‘eukaryotic cell’, a class ‘maximal cellular molecule cluster’, and a class ‘maximal cellular atom cluster’ and all these three classes would have the same extension, although they belong to different frames of reference; and according to the principle of extensionality of class logic, these classes would be identical from a logics point of view. However, from an epistemic point of view, due to the frame- and granularity-dependence of bona fideness, these classes cannot be strictly synonymized [87]. Therefore, when dealing with biological material entities we necessarily have to deal with *multiple cross-granular instantiations* [87] of subcategories of ‘material entity’, all of which do not stand in a subsumption relation to one another. Their requirement is a necessary consequence of the fact that every building block level has its own associated spatio-structural frame of reference.

### Results II: Additional granular perspectives

#### Granular representation and resolution-based representation (RBR) granular perspectives

A consequence of the abovementioned situation of multiple cross-granular instantiation is that each particular biological material entity necessarily instantiates multiple subclasses of ‘material entity’. This can be modeled through providing a URI for each representation. In order to indicate that these URIs refer to the same concrete thing in reality, the resources must be adequately related to one another. Therefore, a specific strict partial ordering relation, i.e., granular representation relation, is introduced, which can be differentiated into *has coarser granular representation* and its inverse relation, *has finer granular representation*. It has ‘spatio-structural entity’ as its range and its domain. This relation gives rise to a granular partition, a *scale-based resolution granular partition*. Scale-based, because the CBB granularity perspective can be interpreted to provide a scale that is based on the ordering of CBB granularity levels from the finest to the coarsest level. *Resolution*, because each individual resource refers to the same concrete material entity, but represents it in its level-specific resolution. This scale-based resolution granular partition also covers the non-countable ‘portion of matter entity’ granular representations of a given particular material entity that can instantiate identical subclasses of ‘portion of matter entity’ across several spatio-structural frames of reference (see Fig. 2c).

As a consequence, the entities that belong to the same scale-based resolution granular partition are only different *granular representations* of the same particular material entity, with each granular representation directly linked to a specific spatio-structural frame of reference [87].

On the basis of this granular representation relation, and in addition to the various qualitative granular perspectives discussed so far, one can differentiate several quantitative scale-based granular perspectives (cf. [58]). This is required to formally model the specific relation between resources that refer to different granular representations of the same particular material entity in various finer and coarser spatio-structural frames of reference.

**Fig. 9:**
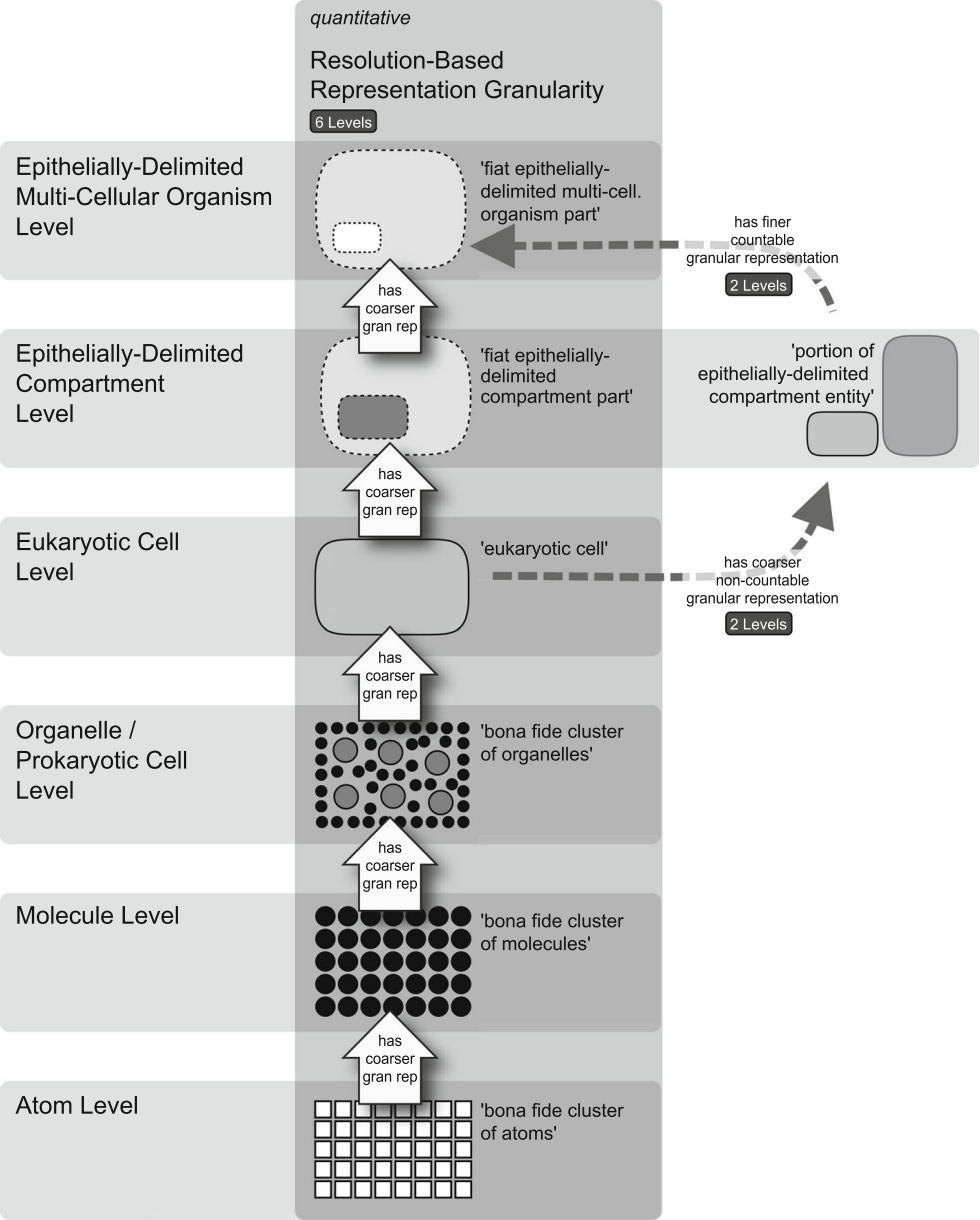
Resolution-Based Representation (RBR) and Resolution-Based Countability Representation (RBCR) Granularity Perspective. The different levels of the RBR granular perspective are granulated according to the *has coarser granular representation* relation (the white broad arrows). The granulation is of the scale dependent grain-size-according-to-resolution granularity type (*sgrG* [61]). The two levels of each of the two RBCR granular perspectives, on the other hand, are granulated according to the *has coarser non-countable granular representation* relation and the *has finer countable granular representation* relation, respectively (dotted gray arrows). Their granulation is of the scale dependent grain-size-according-to-resolution granularity type (*sgrG* [61]). All three perspectives use the combination of the granulation relation together with the scale provided through the set of different spatio-structural frames of reference that are sequentially ordered through the associated CBB granular perspective (i.e., the building block levels hierarchy). As a consequence, the RBR granular perspective comprises six granularity levels, whereas the two RBCR granular perspectives each comprise only two granularity levels, because their granulation relation is *not* transitive (its domain and range differ)

All resolution-based representation (RBR) granular perspectives are based on the combination of the CBB granular perspective and a strict partial ordering granular representation relation between instances of different subclasses of ‘spatio-structural entity’ that belong to different spatio-structural frames of reference. The possibilities for distinguishing different types of RBR granular perspectives is extensive and results from the different range and domain combinations for the granulation relation, with each unique combination resulting in a unique granulation criterion. Here, however, I will only discuss the most general and inclusive type of RBR granular perspective that has the *granulation criterion* (Fig. 9):

X = a specific spatio-structural frame of reference; X + 1 = the next coarser spatio-structural frame of reference adjacent to X.

This perspective has a granulation of the scale dependent grain-size-according-to-resolution *granularity type* (*sgrG* [61]). It is based on the granular representation relation as its *granulation relation*. Because this RBR granular perspective directly depends on the CBB granular perspective, the number of its granularity levels corresponds with the number of CBB granularity levels.

**Table:** 

‘spatio-structural entity’ ^X^	hasCoarserGranRep	‘spatio-structural entity’ ^X + 1^;
‘spatio-structural entity’ ^X + 1^	hasFinerGranRep	‘spatio-structural entity’ ^X^;

#### Resolution-based Countability representation (RBCR) granular perspectives

The RBR granular perspective does not differentiate whether a representation is of the countable building block level entity kind (e.g., ‘atom level entity’, ‘molecule level entity’) or the non-countable ‘portion of matter entity’ kind, as it allows all kinds of spatio-structural entities to be granulated. In order to identify changes from countable to non-countable representations of a given real entity across different spatio-structural frames of reference, two complementary resolution-based countability representation (RBCR) granular perspectives are suggested. For this reason the following two granular countability representation relations are introduced: (i) *has coarser non-countable granular representation* (co_n-c_GranRep), with some building block level entity (e.g., ‘eukaryotic cell level entity’) as its domain and ‘portion of matter entity’ as its range, together with its inverse relation *has finer countable granular representation* (fi_c_GranRep), and (ii) *has coarser countable granular representation* (co_c_GranRep), with ‘portion of matter entity’ as its domain and some building block level entity as its range, together with its inverse relation *has finer non-countable granular representation* (fi_n-c_GranRep). On the basis of these two relations two complementary RBCR granular perspectives can be distinguished: (i) *countable to non-countable RBCR granular perspective*, and (ii) *non-countable to countable RBCR granular perspective*. The countable to non-countable perspective has the *granulation criterion* (Fig. 9):

The non-countable to countable perspective has the *granulation criterion*:

X = a specific spatio-structural frame of reference; X + 1 = the next coarser spatio-structural frame of reference adjacent to X.

These two complementary perspectives have both a granulation of the scale dependent grain-size-according-to-resolution *granularity type* (*sgrG* [61]). Each is based on its respective granular countability representation relation as its *granulation relation*. Because the domain and range of their respective granulation relation differ, the granulation relation is *not* transitive and thus both RBCR granular perspectives comprise only two distinct granularity levels.

**Table:** 

‘spatio-structural entity’ ^X^	co_n-c_GranRep	‘portion of matter entity’ ^X + 1^;
‘portion of matter entity’ ^X + 1^	fi_c_GranRep	‘spatio-structural entity’ ^X^;

**Table:** 

‘portion of matter entity’ ^X^	co_c_GranRep	‘spatio-structural entity’ ^X + 1^;
‘spatio-structural entity’ ^X + 1^	fi_n-c_GranRep	‘portion of matter entity’ ^X^.

#### Function-based representation (F-BR) and historical/evolution-based representation (H/E-BR) granular perspectives

The functional frame of reference requires its own granular representation due to cross-granular multiple instantiation (analogue to cross-granular multiple instantiation as a consequence of multiple spatio-structural frames of reference). This function-related granular representation is required because some instances of ‘spatio-structural entity’ are at the same time also instances of ‘functional unit’. The filter apparatus of a terminal cell of a protonephridium, for instance, instantiates ‘fiat eukaryotic cell part’, because the filter apparatus consists of the cell’s cilium, a filter and a set of microvilli, but not the other parts of the terminal cell. The filter apparatus, however, also instantiates ‘functional unit’, because it functions as a filter during excretion.

The historical/evolutionary frame of reference also requires its own granular representation due to cross-granular multiple instantiation. Every anatomical entity that is a homologue and that thus instantiates ‘historical/evolutionary unit’ also instantiates ‘spatio-structural entity’.

For this reason the following two granular representation relations are introduced: (i) *has functional granular representation* (FuncGranRep), with ‘spatio-structural entity’ as its domain and ‘functional entity’ as its range and its inverse relation *functional has spatio-structural granular representation* (FuncSp-StrGranRep), and (ii) *has historical/evolutionary granular representation* (Hist/EvGranRep), with ‘spatio-structural entity’ as its domain and ‘historical/evolutionary entity’ as its range and its inverse relation *historical/evolutionary has spatio-structural granular representation* (Hist/EvSp-StrGranRep). On the basis of these two relations two granular perspectives can be distinguished: (i) a *function-based representation (F-BR) granular perspective* and (ii) a *historical/evolution-based representation (H/E-BR)* granular perspective. The F-BR granular perspective has the *granulation criterion*:

The H/E-BR granular perspective has the *granulation criterion*:

These two perspectives have both a granulation of the scale-dependent grain-size-according-to-resolution *granularity type* (*sgrG* [61]). *Resolution* is here used in the sense of depending on a specific frame of reference that functions like a lens for filtering out all aspects irrelevant to the given frame of reference. Each is based on its respective granular representation relation as its *granulation relation*. Because in both perspectives the domain and range of the respective granulation relations differ, the granulation relations are *not* transitive. Consequently, both granular perspectives comprise only two distinct granularity levels.

**Table:** 

‘spatio-structural entity’	FuncGranRep	‘functional entity’;
‘functional entity’	FuncSp-StrGranRep	‘spatio-structural entity’.

**Table:** 

‘spatio-structural entity’	Hist/EvGranRep	‘historical/evolutionary entity’;
‘historical/evolutionary entity’	‘Hist/EvSp-StrGranRep	‘spatio-structural entity’.

## Discussion

The here proposed approach for the development of a domain granularity framework for the life sciences comprises a core set of granular perspectives that can be utilized for efficiently managing large semantic graphs that contain data about material entities that range from atoms to multi-cellular organisms and beyond. The granularity framework provides a meta-layer that (i) defines the relations between entities that belong to different granularity levels of the same granular perspective and between entities across different granular perspectives; (ii) integrates various frames of reference within a single framework, all of which are essential for the life sciences, ranging from purely spatio-structural frames of reference, to functional, developmental, ecological, and evolutionary frames of reference; (iii) improves searching and navigating through large complex graphs by using one or a combination of several granular perspectives as filters and for efficiently utilizing the hierarchical structure inherent in the semantic graphs; and (iv) facilitates reasoning and inferencing by providing additional hierarchical structures that can be used for measuring semantic similarities *between* different semantic graphs and between resources *within* a graph.

This domain granularity framework complies with Craver’s [23] claim of descriptive pluralism about the levels idea. It comprises various hierarchies of different levels. The compositional building block (CBB) granular perspective (Fig. 4) takes in a key position in the framework, because it provides the backbone hierarchy that facilitates the integration of all the other granular perspectives. The CBB granular perspective resembles a purely compositional account of the levels idea, without making the mistake to mix entities relevant in different frames of reference (see problems discussed further above regarding Eldredge’s somatic hierarchy [9]). Furthermore, with its focus on physical covering and evolving building blocks, the CBB granular perspective is also influenced by the evolutionary systems-theoretical accounts of the levels idea, thereby integrating purely spatio-structural considerations with functional and evolutionary aspects. The set of region-based granular perspectives, on the other hand, do not have a pre-defined structure in terms of a fix number of granularity levels, but must be determined on a local case-by-case approach, thereby reflecting one of the criticism regarding the single compositional hierarchy of the compositional account of the levels idea (for the compositional account of levels see [4, 29, 33, 117, 128, 129]; for critique of this approach see [44, 130–133]).

The set of functional parthood-based granular perspectives resemble the mechanism-based account of the levels idea [119–123]. The lack of a globally applicable general granular perspective comparable to the CBB granular perspective for functional parthood thereby reflects that functional parthood-based granularity levels depend on a given mechanism (i.e., a function, and therefore also a causal process) and thus are local, case-specific, and cannot result in a universal scheme that is globally applicable [120]. And finally, the different spatio-structural frames of reference, with their diverse sets of parthood-based granular perspectives, together with the granular perspectives mediating between these and other frames of reference, reflect many aspects that Wimsatt [4, 35, 117, 134] discussed in his prototypical account of levels of organization.

Although this domain granularity framework for the life sciences comprises all these different accounts of the levels idea, it nevertheless is characterized and defined in a formally coherent framework that integrates all these diverse granular perspectives. There might be conceptually and computationally simpler and more elegant solutions to the theoretical, conceptual, and computational challenge of modeling the granularity of cumulative-constitutively organized (biological) material entities, but these solutions model the hierarchies found in reality incorrectly. It seems that if we want to do justice to the complex nature of reality, our models must be complex as well.

## Conclusion

A domain granularity framework based on Keet’s theory of granularity would not only provide a much needed conceptual framework for representing domains that cover multiple granularity levels such as anatomy/morphology or the life sciences in general, but also a structure that can be utilized for providing users a more intuitive experience when navigating and exploring data represented as semantic graphs in knowledge bases and content management systems of the life sciences. The framework could, for instance, be used for querying a given semantic graph in order to retrieve any partition expressed in the graph that corresponds with the granular perspective that the user is interested in. The framework can contain various such perspectives, each of which can be applied on a given semantic graph or knowledge base to the effect of filtering out all information irrelevant to this particular perspective, thereby substantially facilitating a desperately needed system that supports browsing and navigating through increasingly complex semantic graphs (i.e., datasets).

If the hierarchical order of the various granular perspectives contained in a domain granularity framework reflects reality, the framework would provide a hierarchical structure that could be meaningfully employed for reasoning over different granularity levels and even different granular perspectives, thereby providing a methodological basis for effectively establishing comparability between different semantic graphs, which can be used for automatic assessment and measurement of *semantic similarity* between different semantic graphs. Being able to quantitatively measure degrees of similarity between semantic graphs would provide new means for analyzing all kinds of data from the life sciences (e.g., [135–137].

## Abbreviations

BFO: Basic Formal Ontology
CBB: Compositional Building Block
CBB-C: Compositional Building Block Cluster
CFU: Compositional Functional Unit
CH/EU: Compositional Historical/Evolutionary Unit
ChIN: Character Identity Network
co_c_GranRep: Has-Coarser-Countable-Granular-Representation relation
co_n-c_GranRep: Has-Coarser-Non-Countable-Granular-Representation relation
DirPropHistEvolPartOf: Direct-Proper-Historical/Evolutionary-Part-Of relation
ECM: Extracellular matrix
evo-devo: Evolutionary developmental biology
F-BR: Function-Based Representation
fi_c_GranRep: Has-Finer-Countable-Granular-Representation relation
fi_n-c_GranRep: Has-Finer-Non-Countable-Granular-Representation relation
FuncGranRep: Has-Functional-Granular-Representation relation
FuncSp-StrGranRep: Functional-Has-Spatio-Structural-Granular-Representation relation
H/E-BR: Historical/Evolution-Based Representation
hasCoarserGranRep: Has-Coarser-Granular-Representation relation
hasDirPropHistEvolPart: Has-Direct-Proper-Historical/Evolutionary-Part relation
hasFinerGranRep: Has-Finer-Granular-Representation relation
Hist/EvGranRep: Has-Historical/Evolutionary-Granular-Representation relation
Hist/EvSP-StrGranRep: Historical/Evolutionary-Has-Spatio-Structural-Granular-Representation relation
npG: Non-scale dependent primitive granularity type
nrG: Non-scale dependent single-relation-type granularity type
OBO Foundry: Open Biomedical Ontologies Foundry
RBCR: Resolution-Based Countability Representation
RBR: Resolution-Based Representation
sgrG: Scale dependent grain-size-according-to-resolution granularity type
URI: Uniform Resource Identifier
